# Methylome-wide and meQTL analysis helps to distinguish treatment response from non-response and pathogenesis markers in schizophrenia

**DOI:** 10.3389/fpsyt.2024.1297760

**Published:** 2024-03-07

**Authors:** Binithamol K. Polakkattil, Neetha N. Vellichirammal, Indu V. Nair, Chandrasekharan M. Nair, Moinak Banerjee

**Affiliations:** ^1^ Human Molecular Genetics Laboratory, Rajiv Gandhi Centre for Biotechnology, Thiruvananthapuram, Kerala, India; ^2^ Research Center, University of Kerala, Thiruvananthapuram, Kerala, India; ^3^ Mental Health Centre, Thiruvananthapuram, Kerala, India; ^4^ Nair’s Hospital, Maradu, Kerala, India

**Keywords:** schizophrenia, epigenetics, methylation, SNP genotyping, gene expression, treatment response, antipsychotics

## Abstract

Schizophrenia is a complex condition with entwined genetic and epigenetic risk factors, posing a challenge to disentangle the intermixed pathological and therapeutic epigenetic signatures. To resolve this, we performed 850K methylome-wide and 700K genome-wide studies on the same set of schizophrenia patients by stratifying them into responders, non-responders, and drug-naïve patients. The key genes that signified the response were followed up using real-time gene expression studies to understand the effect of antipsychotics at the gene transcription level. The study primarily implicates hypermethylation in therapeutic response and hypomethylation in the drug-non-responsive state. Several differentially methylated sites and regions colocalized with the schizophrenia genome-wide association study (GWAS) risk genes and variants, supporting the convoluted gene–environment association. Gene ontology and protein–protein interaction (PPI) network analyses revealed distinct patterns that differentiated the treatment response from drug resistance. The study highlights the strong involvement of several processes related to nervous system development, cell adhesion, and signaling in the antipsychotic response. The ability of antipsychotic medications to alter the pathology by modulating gene expression or methylation patterns is evident from the general increase in the gene expression of response markers and histone modifiers and the decrease in class II human leukocyte antigen (HLA) genes following treatment with varying concentrations of medications like clozapine, olanzapine, risperidone, and haloperidol. The study indicates a directional overlap of methylation markers between pathogenesis and therapeutic response, thereby suggesting a careful distinction of methylation markers of pathogenesis from treatment response. In addition, there is a need to understand the trade-off between genetic and epigenetic observations. It is suggested that methylomic changes brought about by drugs need careful evaluation for their positive effects on pathogenesis, course of disease progression, symptom severity, side effects, and refractoriness.

## 1 Introduction

Schizophrenia is a severe neuropsychiatric condition with a lifetime weighted prevalence of 0.6% and a current weighted prevalence of 0.12% among the population of Kerala (1). This condition is characterized by diverse positive, negative, and cognitive symptoms resulting from a complicated interaction between genetic and environmental factors (2). Antipsychotic drugs are extensively used for reducing the symptomatic burden of psychosis. Research indicates that insufficient clinical intervention in the early stages of psychosis raises the mortality rate among young individuals (3). Even when antipsychotic therapy was considered a primary treatment, refractoriness to medication was observed in 23% of the patients (4, 5). Treatment resistance is the persistence of symptoms after two medication trials with appropriate dosage, duration, and proper adherence (6). Clozapine is regarded as the primary pharmacological strategy, but the adverse effects associated with clozapine have raised concerns over its use (7). In patients unresponsive to clozapine treatment, atypical antipsychotic polypharmacy is suggested as an alternative, albeit with the potential drawback of metabolic syndrome and other side effects (8, 9). Studies have indicated the use of quetiapine over clozapine to treat individuals with significant negative symptoms due to clozapine’s adverse effects (10, 11). Therefore, identifying markers contributing to the positive response or lack of response helps in the early prediction of treatment outcomes to avoid the impact of multiple drug treatments.

Genome-wide association study (GWAS) conclusively demonstrates the heritability of schizophrenia contributed by multiple variants that were predominantly spread across genes expressed in the central nervous system neurons (12). These risk loci and their risk alleles do demonstrate differential expression that can further be influenced by environmental variables and thereby may play a role in the etiology of schizophrenia. Gene expression is tightly regulated, and epigenetic processes such as DNA methylation can influence it by altering chromatin composition (13). The gene regulation through epigenetic mechanisms is responsive to the environmental risk variables linked to the development of schizophrenia (13–15). The decreased concordance rate in monozygotic twin studies corroborates the involvement of environmental risk factors (16, 17). Genome-wide methylation variations have been extensively studied in association with schizophrenia in several surrogate and brain tissues (18–21), and these associated differentially methylated positions strongly colocalized with schizophrenia risk loci, further supporting the intertwined genetic and epigenetic regulation of multifactorial disorders (22). Evidence also implies that antipsychotic drugs can affect the epigenome (23–26).

In general, large cohort studies give less emphasis on antipsychotic treatment response; therefore, disease-associated methylation markers are likely to be confounded by drug-induced alterations. Studies have indicated that antipsychotic drugs could mire these epigenetic observations; in addition, the schizophrenia pool could also comprise of drug response and resistant pool (24). In a conventional study protocol, it is difficult to have monotherapy patients in assessing the epigenetic landscape. Therefore, in the current study, we aimed to identify the DNA methylation signatures of treatment response and resistance in an ethnically stratified population (27, 28) by examining aberrations in the DNA methylation level of drug-naïve patients, treatment responders, and treatment non-responders using whole blood DNA.

## 2 Materials and methods

### 2.1 Study subjects for DNA methylation study

Blood samples from a Malayalam-speaking Kerala population, with 48 participants diagnosed with schizophrenia, were collected using the *Diagnostic and Statistical Manual of Mental Disorders* (DSM)-IV criteria. The participants were divided into treatment responders (n = 20) and non-responders (n = 20) based on their Brief Psychiatric Rating Scale (BPRS) score improvement after 1 year of follow-up as described earlier (29). All patients received multidrug therapy except for drug-naïve participants (n = 8). The drugs prescribed to the study subjects were olanzapine, clozapine, haloperidol, and risperidone. The average antipsychotic dose was converted to CPZeq. The responders were given an average CPZeq of 537.21 ± 48.37 mg/day, while non-responders were given 622.22 ± 109.8 mg/day. Demographic information of the study samples is listed in **Supplementary Table S1**. Except for the drug-naïve participants, all other participants’ sampling was conducted after confirming their treatment response status. DNA was isolated from peripheral blood using the phenol–chloroform method. The Institutional Ethics Committee approved the study for biomedical subjects as per the Indian Council of Medical Research (ICMR) and Helsinki protocol.

### 2.2 DNA methylation analysis

**Figure 1:**
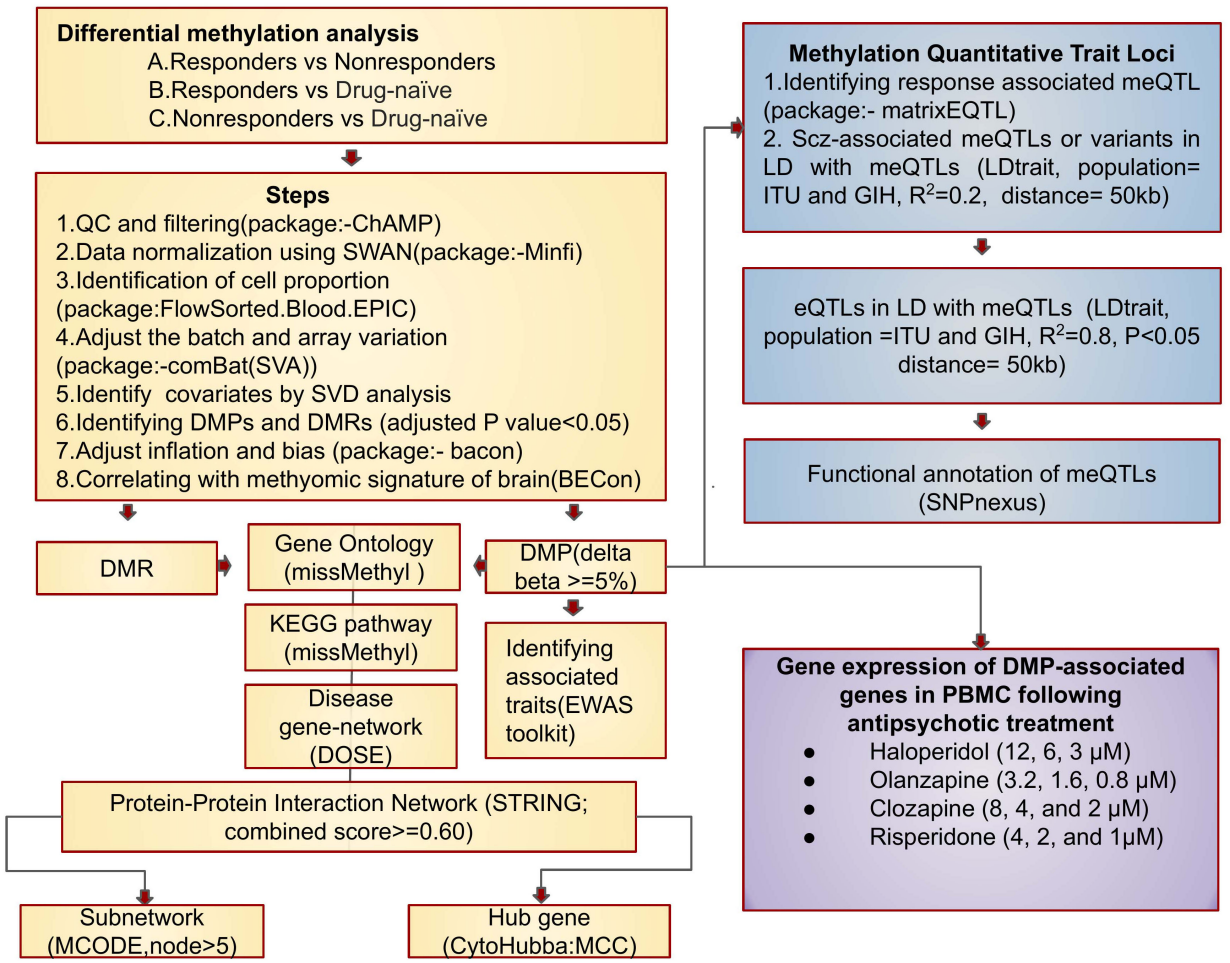
Flowchart detailing the study workflow.

To investigate the DNA methylation patterns in drug-naïve, treatment responder, and non-responder schizophrenia patients, the Illumina Methylation EPIC 850K BeadChip (Illumina, San Diego, CA, USA) was used. The whole blood DNA was bisulfite-converted using the EZ DNA Methylation™ kit (Zymo Research, Irvine, CA, USA) following the standard protocol. The converted DNA was amplified and enzymatically fragmented before being applied to the array for hybridization, followed by a single-base extension. The Illumina iScan system was used to scan the fluorescently labeled BeadChip. The intensity data files from Illumina were imported into the R programming environment version 4.1.0 (30) and analyzed using the ChAMP v2.28.0 package (31, 32). The following were excluded: probes that had a detection p-value greater than 1e−16 and failed p-value threshold above 0.01, probes with a bead count of less than 3 in more than 5% of the samples, non-CpG probes, probes located near single-nucleotide polymorphisms (SNPs) defined by their allele frequency in Indian Telugu in the UK (ITU) population data, cross-reacting probes provided by maxprobes v0.0.2 R package, and sex chromosome probes. Samples with a failed p-value above the proportion of the NA ratio threshold of 5% were filtered out. The list of probes filtered out from the study is provided in **Supplementary Table S2**. One sample from the non-responder category was eliminated based on the discrepancy between the estimated and reported sex. On filtered probes, the SWAN normalization technique was applied (33). The “estimateCellCounts2” function in the FlowSorted.Blood.EPIC v2.2.0 package was used for cell count estimation (**Supplementary Figure S1**) (34). The comBat batch correction accounted for array and batch variations (35). Using the “champ.SVD” function, the confounding variables were identified and regressed. The flowchart of the analysis is shown in **Figure 1**.

### 2.3 Identification of differentially methylated probes

The DNA methylation level was quantified as a beta score. The function “champ.DMP” uses the limma package to pairwise compare the phenotype of interest to identify differentially methylated areas (36). Potential inflation and bias were controlled using the R package Bacon v1.26.0 (37). The conservative Bonferroni method was employed to control the family-wise error rate (FWER) (p < 0.1) and the Benjamini and Yekutieli method (p < 0.05) to control the false discovery rate (FDR). Additionally, the Psychiatric Genomics Consortium-EWAS pipeline was utilized to enhance the sensitivity of our findings (38). The significance of the overlap between the comparisons was evaluated using Fisher’s exact test, and the similarity between the probe list was defined in terms of the Jaccard index using the R package GeneOverlap v1.38.0 and HelloRanges v1.27 (39, 40). BECon was used to determine the relationship between the differences in CpG sites between surrogate tissue, blood, and the brain (41). Using the EWAS-atlas platform, a correlation between probe methylation and a trait was inferred (42).

### 2.4 Identification of the differentially methylated region

The Bumphunter algorithm through the “champ.DMR” function was used to estimate the difference in DNA methylation with regard to a genomic region (43). The minimum number of probes per differentially methylated region (DMR) was set at 7, while the maximum length of the DMR was set at 300. Alternatively, the DMRcate algorithm was also used to estimate DMRs (44). The array manifest file was used to annotate all DMRs above the p-value limit of 0.05. Assessment of the functional role of the top 1,000 DMRs was made through the gene ontology (GO) enrichment and Kyoto Encyclopedia of Genes and Genomes (KEGG) pathway analyses using the DMR annotation tool provided by the scMethBank (45). Using the DOSE v3.28.1 package function “enrichDGN”, all the publicly available disease–gene associations listed in the DisGeNET platform were probed, and an overrepresentation analysis among the differentially methylated genes was performed (46).

### 2.5 Functional enrichment analysis of DMPs

The “gometh” function from the missMethyl v1.32.0 package was used for differentially methylated probe (DMP) enrichment analysis of all probes with |Δβ| > 0.05 to eliminate bias resulting from the presence of varying numbers of CpG probes per gene (47). The Benjamini and Hochberg method was used to adjust p value the significance level was set to 0.05.

### 2.6 Protein–protein interaction network of DMP- and DMR-associated genes

A protein–protein interaction network of genes associated with DMPs (|Δβ| ≥ 0.05), as well as the DMRs, was constructed using the STRING database, and the network was loaded into Cytoscape v3.9.1 to identify hub genes using the CytoHubba plugin (combined score >0.6) (48–50). The MCODE plugin’s default parameters were utilized to discover dense gene subnetworks (degree cutoff = 2, mode score cutoff = 5, max.depth = 100, k score = 2) among the DMP and DMR genes (51).

### 2.7 SNP genotyping and methylation QTL analysis

Infinium Global Screening Array-24 v3.0 BeadChip comprising 700k genotypes was used to screen in genomic DNA isolated from the whole blood of responders (n = 18) and non-responders (n = 16) (Illumina, USA) following the standard protocol. Denatured DNA underwent enzymatic fragmentation, followed by hybridization with a bead chip. Hybridized primers were then extended and stained for detection and analysis. Finally, the BeadChips were scanned using Illumina iScan to generate the Illumina Intensity Data files or IDAT. The IAAP Genotyping Command Line Interface (CLI) generated vcf files using Illumina intensity data. Quality control was performed using the R package plinkQC v0.3.4 (52), omitting samples with >1% missing variants and SNPs with >1% missingness, Hardy–Weinberg equilibrium p-value <0.05, and a minor allele frequency of 5%. Variants on chromosomes 1–22 were only considered for this investigation. The probes that were excluded from the analysis are shown in **Supplementary Table S2**. R package MatrixEQTL v2.3 was used to identify the association between SNP–methylation probe pairs; sex, age, batch, slide, cell type composition, and principal components (first 10) from the genotype data were included as covariates (53). All significant DMPs were examined for association with SNPs. All the SNPs located within 50 kb of the CpG sites were considered *cis*–methylation quantitative trait locus (meQTL), and the significance threshold was set as FDR ≤ 0.1. The meQTL variants or variants in linkage disequilibrium (LD) within them were searched for reported association with the disease phenotype: schizophrenia (population-GIH, ITU; R′22 ≥ 0.2; 50-kb base pair window), and association to tissue-specific gene expression (blood; population-GIH, ITU; R′2 ≥ 0.8; p < 0.05; 50-kb base pair window) were enquired using the LDlink suite (54). Colocalization analysis was performed for the *cis*-meQTLs and treatment response GWAS variants using the coloc package v5.2.2 (55).

### 2.8 Peripheral blood mononuclear cell culture and antipsychotic treatment

Peripheral blood mononuclear cells (PBMCs) were isolated from 10 ml of blood obtained from a healthy donor by density gradient centrifugation using a low-viscosity medium (HiSepTM LS 1077, HiMedia, Thane, India). Blood was diluted (1:1) with 1× phosphate-buffered saline (PBS) carefully placed over a low-viscosity medium and centrifuged at 750 g for 30 min at 27°C. The PBMC-containing buffy coat was removed and washed with 1× PBS 350 g for 10 min. PBMC was suspended in 1× red blood cell (RBC) lysis buffer for 5 min to eliminate any residual RBC from the separation procedure. The PBMC pellets were rinsed twice with 1× PBS buffer and resuspended in RPMI-1640 media with 2 mM l-glutamate (HiMedia), antibiotics (1× penicillin/streptomycin, Thermo Scientific, Waltham, MA, USA), and 10% heat-inactivated fetal bovine serum (Gibco, Grand Island, NY, USA). Viable cells were plated at a density of 2 * 10^6^/ml in a 6-well plate and cultured for 72 hours at 37°C and 5% CO_2_ with different concentrations of the following antipsychotic drugs (Sigma): haloperidol (12 μM, 6 μM, and 3 μM), olanzapine (3.2 μM, 1.6 μM, and 0.8 μM), clozapine (8 μM, 4 μM, and 2 μM), and risperidone (4 μM, 2 μM, and 1 μM). The concentration of antipsychotic medicine used for the drug treatment was based on an earlier study (56). The drugs were dissolved in dimethyl sulfoxide (DMSO), and 0.1% DMSO-treated cells were used as the assay control in our study. After 72 hours of drug treatment, cells were pelted for RNA extraction.

### 2.9 Gene expression study

Total RNA was extracted from the PBMCs with the TRIzol reagent (Invitrogen, Carlsbad, CA, USA: 15596026) and treated with “RQ1 RNase-Free DNase” (Promega, Madison, WI, USA: M6101) according to the manufacturers’ protocol. Following the quantification using NanoDrop (Thermo Scientific), 1 µg of RNA was reverse transcribed to cDNA using the “PrimeScript 1st Strand cDNA Synthesis Kit” (Takara Bio, Mountain View, CA, USA: 6110A) based on the manufacturer’s instructions. A SYBR-based assay using the “TB Green Premix Ex Taq II (Tli RNaseH Plus)” kit (Takara Bio: RR820W) was used to estimate the mRNA level. A 5-μl reaction was performed with “1× TB Green Premix Ex Taq II (Tli RNaseH Plus)” mix, 0.4 μM forward and reverse primers, 1× ROX reference dye, RNase-free water, and 5 ng of RNA. The National Center for Biotechnology Information (NCBI) primer blast and IDT primer designing software were used to design primers. For each sample, triplicates were employed for running qRT-PCR experiments in Quant Studio 5 (Applied Biosystems, Foster City, CA, USA) as per the manufacturer’s protocol: 95°C for 3 min, followed by 40 cycles with 95°C for 5 s, 60°C for 34 s at a ramp rate of 1.6°C/s. Conditions for the melt curve were as follows: 95°C for 15 s, 60°C for 1 min at 1.6°C/s, and 95°C for 0.01 s at a ramp rate of 0.15°C/s. The endogenous control β-actin was used to normalize gene expression, and its relative expression was determined using the comparative Ct (2^−ΔΔCt^) method. Sequence details of primers used in this study are listed in **Supplementary Table S3**. Differences between groups were evaluated by one-way ANOVA with Sidak’s multiple-comparison test using GraphPad prism software v8.1, and the data are presented as mean ± standard error of the mean of two independent experiments.

## 3 Results

### 3.1 Differential DNA methylation analysis

**Figure 2:**
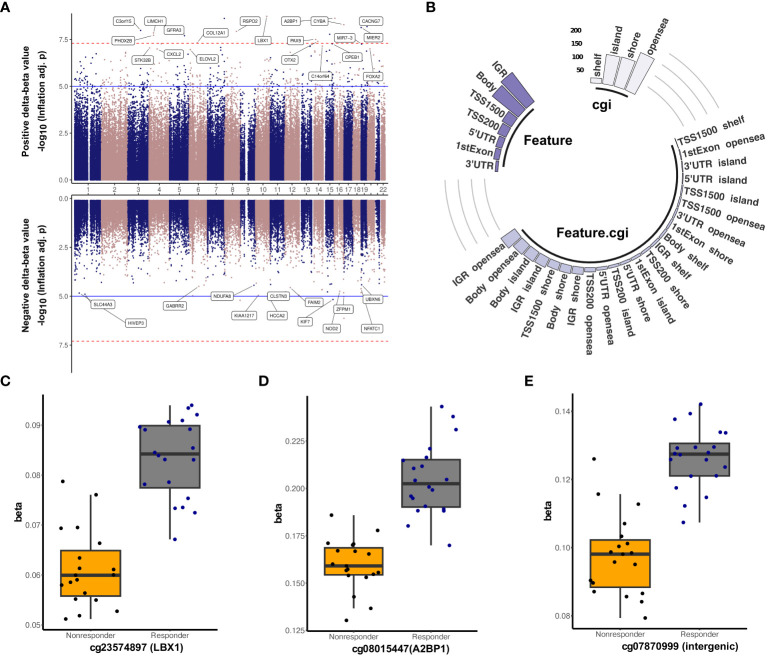
Differentially methylated sites marking treatment response. **(A)** Miami plot illustrating differentially methylated probes between antipsychotic treatment responders and non-responders. The top panel represents the probes with a positive delta-beta value (|Δβ|) (hypermethylated), while the bottom panel presents the probes with a negative delta-beta value (hypomethylated). The blue line represents the p-value cutoff of 1e−05 (suggestive line), and the dashed red line denotes the p-value cutoff of 5e−8 (genome line). The top 20 CpG sites are labeled except for those probes located in the intergenomic region. **(B)** A circular bar plot depicting the distribution of significant CpG sites differentially methylated between responders and non-responders, with |Δβ| ≥ 5% into different genomic region features such as transcription start site (200 and 1,500 bp from start site), 1stExon, gene body, intergenomic region, 5′ and 3′ untranslated regions and different CpG island (CGI) classifications like island, open sea, shore, and shelf. Boxplot of the top three significant differentially methylated sites: **(C)** cg23574897, **(D)** cg08015447, and **(E)** cg07870999 associated with response.

**Figure 3:**
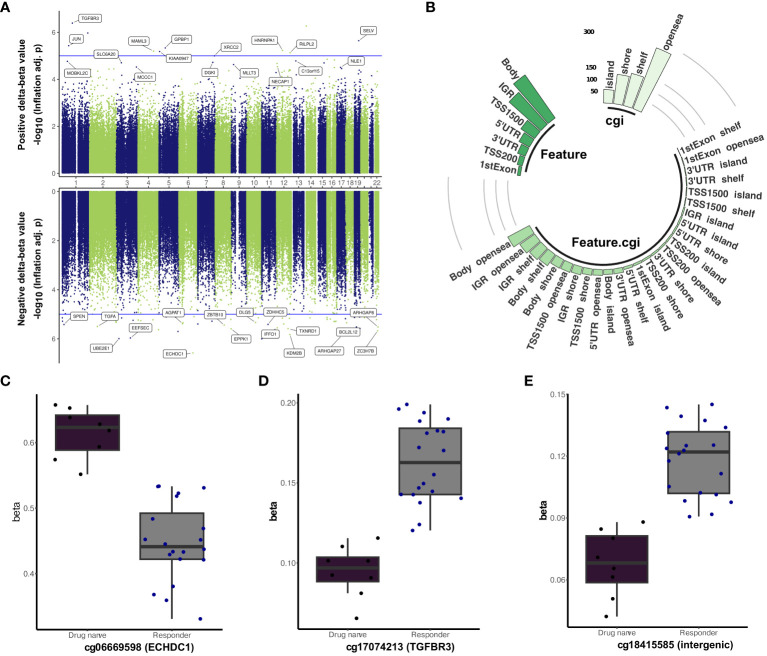
Differentially methylated sites marking treatment effectiveness. **(A)** Miami plot depicting the differentially methylated probes between antipsychotic responders and drug-naïve. The top and bottom panels represent positive and negative delta-beta values, respectively. The blue line indicates the p-value cutoff of 1e−05 (suggestive line), while the dashed red line represents the p-value cutoff of 5e−8 (genome line). **(B)** A circular bar plot showing the distribution of differentially methylated CpG sites between responders and drug-naïve patients with mean methylation difference above 5%. Boxplot of the top-ranked CpG markers linked with treatment effectiveness: **(C)** cg06669598, **(D)** cg17074213, and **(E)** cg18415585.

**Figure 4:**
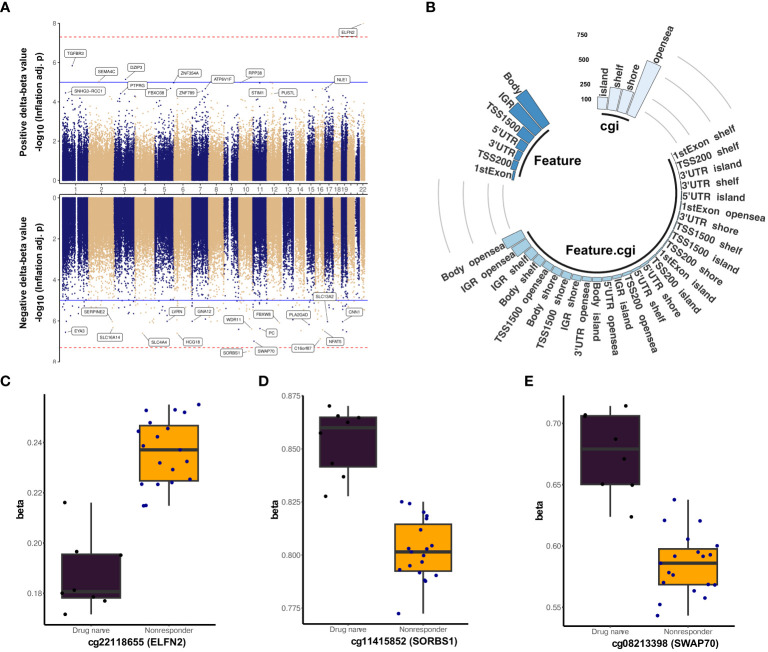
Differentially methylated sites marking treatment resistance. **(A)** Miami plot of differentially methylated sites identified from non-responders and drug-naïve comparison. The top and bottom panel denotes positive and negative delta-beta values, respectively. The blue line indicates the p-value cutoff of 1e−05 (suggestive line), and the red line represents the p-value cutoff of 5e−8 (genome line). **(B)** Distribution of differentially methylated CpG sites between non-responders and drug-naïve with mean methylation difference above 5% are represented in a circular bar plot. Boxplot showing the top three CpG markers of treatment resistance: **(C)** cg22118655, **(D)** cg11415852, and **(E)** cg08213398.

The DNA methylation levels differentiating drug responders and drug non-responders are referred to as markers of treatment response (**Figure 2**), those between drug responders and drug-naïve patients are referred to as markers of treatment effectiveness (**Figure 3**), and those between drug-naïve patients and drug-non-responders are referred to as markers of treatment resistance (**Figure 4**). **Table 1** details the number of differential sites identified in various comparisons.

**Table 1:** Distribution pattern of the significant differentially methylated sites identified in various comparisons.

Category	Responders–non-responders	Responders–drug naïve	Non-responders–drug naïve
No. of probes after filtration	726,496	737,506	728,374
Number of significant DMPs	10,612	4,659	7,928
Hypermethylated	10,436	452	722
Hypomethylated	176	4,207	7,206
Number of significant DMPs with (Δβ) > 0.05	398	592	1161

DMPs, differentially methylated probe; Δβ, average methylation difference between two groups.

#### 3.1.1 Methylation signatures differentiating the treatment responders and non-responders

Differential DNA methylation analysis following the adjustment of covariates such as age, sex, count of B cell, neutrophil, CD8T, natural killer, and monocytes (**Supplementary Figure S1A**) we identified 10,612 differentially methylated probes (FDR-adjusted p-value <0.05) (**Table 1**, **Figures 2A, B** and **Supplementary Table S4**) with an inflation of 1.4 and bias of 0.35 (**Supplementary Figure S2**). After the Bonferroni correction, 380 CpG sites remained significant, and the top three sites were cg23574897 (*LBX1*, TSS200, |Δβ| = 0.02, Bonferroni adj. p-value = 9.62E−05), cg08015447 (*A2BP1*, 5′UTR, |Δβ| = 0.04, Bonferroni adj. p-value = 1.13E−04), and cg07870999 (intergenic, |Δβ| = 0.03, Bonferroni adj. p-value = 1.14E−04) (**Figures 2C–E**). Among them, 398 loci exhibited a mean methylation difference (Δβ) greater than 5%, and the distribution pattern of these selected loci revealed enrichment within the open sea region (38.44%) as shown in **Figure 2B**. Probe distribution displayed a higher prevalence in the intergenomic region (34.92%) compared to the gene body (31.91%) and promoter (24.62%), with a predominant localization on chromosome 1 (12.56%). Furthermore, a substantial proportion of probes (n = 396) displayed significantly elevated DNA methylation levels in treatment responders. The probe, cg08617160, situated within the promoter region of the transcriptional repressor gene *MIER2* exhibited the most significant hypermethylation, evidenced by its inflation-adjusted p-value of 7.24E−09. Following in significance were cg00630212 in the promoter of inflammatory gene *CXCL2* (Inflation adj. p-value = 1.01E−07) and cg21184711 within the gene body of *CADPS2* (Inflation adj. p-value = 1.18E−07) (**Supplementary Figures S3A–E**). Notably, seven hypermethylated probes were mapped to the genomic coordinates of the filamin A binding protein-encoding gene, *FILIP1*, followed by *PM20D1* (n = 6), *GALNT9* (n = 5), *FMOD* (n = 4), and *SLC17A9* (n = 4). The highest group-wise methylation differences were observed for cg20415053 (*CATSPER4*, body, |Δβ| = 0.17), cg19637330 (intergenic, |Δβ| = 0.16), and cg09044981 (*CDH13*, body, |Δβ| = 0.13) (**Table 2**, **Supplementary Figures S4A–C**). Furthermore, enrichment analysis of hypermethylated probes identified associations with specific trait categories, including childhood stress, maternal factors, folate supplementation, ethnicity and gestational events (**Supplementary Table S5**). In contrast, only two hypomethylated sites, cg11309454 annotated to *CCBL2* and cg07679219 associated with *E2F7*, displayed mean DNA methylation differences above 5% (**Supplementary Figures S3D, E**).

**Table 2:** Top differentially methylated positions associated with treatment response, effectiveness, and resistance.

Hypermethylated probes					Hypomethylated probes				
Probe	Δβ	Adj.P.Val	Gene	Feature	Probe	Δβ	Adj.P.Val	Gene	Feature
Responders versus non-responders (treatment response)									
cg20415053	0.17	3.07E−03	*CATSPER4*	Body	cg07679219	−0.13	3.43E−02	*E2F7*	3′UTR
cg19637330	0.16	2.24E−02		IGR	cg11309454	−0.09	1.69E−02	*CCBL2*	TSS1500
cg09044981	0.16	1.96E−02	*CDH13*	Body					
cg09408571	0.15	4.49E−02	*GPR88*	TSS200					
cg19142181	0.15	1.46E−02	*SLC17A9*	Body					
cg08584759	0.15	3.54E−02	*C10orf47*	Body					
cg26987645	0.15	3.56E−02	*FMOD*	TSS200					
cg15000813	0.14	2.06E−02	*PDGFRL*	Body					
cg14159672	0.14	2.68E−02	*PM20D1*	1stExon					
cg19346084	0.14	3.83E−02		IGR					
Responders versus drug-naïve (treatment effectiveness)									
cg01132696	0.14	4.86E−02	*HLA-DPB1*	Body	cg06669598	−0.17	3.32E−02	*ECHDC1*	3′UTR
cg25858983	0.14	3.44E−02	*UBTF*	5′UTR	cg06579481	−0.16	4.00E−02		IGR
cg10861005	0.12	4.71E−02	*RGL2*	Body	cg08938155	−0.15	3.57E−02	*TBCA*	Body
cg12981595	0.10	3.57E−02	*KRTAP4-8*	TSS200	cg23237765	−0.15	3.32E−02	*C7orf20*	Body
cg06928346	0.09	3.79E−02	*GPR19*	1stExon	cg23836814	−0.15	3.32E−02	*AGBL2*	3′UTR
cg14787155	0.09	3.57E−02	*DZIP3*	1stExon	cg09087363	−0.15	4.55E−02		IGR
cg25746764	0.09	3.50E−02	*ACBD3*	5′UTR	cg11641410	−0.15	3.93E−02		IGR
cg03190379	0.09	3.32E−02	*PRPH*	Body	cg10162971	−0.14	4.44E−02	*GRHL1*	Body
cg21040775	0.09	4.49E−02		IGR	cg26332016	−0.14	3.32E−02	*FBXW8*	TSS1500
cg04829186	0.09	3.32E−02		IGR	cg03319894	−0.13	3.32E−02	*ZBTB10*	Body
Non-responders versus drug-naïve (treatment resistance)									
cg10861005	0.12	4.42E−02	*RGL2*	Body	cg00863893	−0.24	3.79E−02	*TIMP2*	Body
cg26220594	0.12	4.55E−02		IGR	cg10317314	−0.19	2.13E−02		IGR
cg12697442	0.11	9.87E−03	*YAP1*	TSS200	cg14687298	−0.18	4.49E−02		IGR
cg02719245	0.10	4.60E−02	*FLJ45244*	Body	cg19142181	−0.18	2.29E−02	*SLC17A9*	Body
cg00106685	0.09	1.48E−02	*GNL3*	1stExon	cg17221813	−0.18	2.43E−02	*SLC17A9*	Body
cg04829186	0.08	1.48E−02		IGR	cg09314196	−0.17	1.95E−02	*ZNF492*	TSS1500
cg01952234	0.08	1.39E−02	*WT1*	TSS200	cg08584759	−0.17	2.57E−02	*C10orf47*	Body
cg07848310	0.08	1.44E−02	*NNT*	5′UTR	cg12489353	−0.17	2.94E−02	*EHD2*	Body
ch.10.907315R	0.08	1.40E−02	*CCNY*	Body	cg17292337	−0.17	2.86E−02		IGR
cg03190379	0.08	8.46E−03	*PRPH*	Body	cg26429022	−0.16	3.45E−02		IGR

Additional details of all the sites identified in these comparisons are given in **Supplementary Tables S4** (response), **Supplementary Table S7** (effectiveness), and **Supplementary Table S9** (resistance).

Probe, name of the CpG sites; Δβ, difference in the average methylation value between comparing groups; adj.P.Val, p-value adjusted using the Benjamini and Yekutieli method; gene, gene associated with the differentially methylated probe (DMP); feature, genomic features associated with probes.

Listed are the top 10 significant (adjusted p-value >0.05) hyper- or hypomethylated sites between (1) responders and non-responders (treatment response), (2) responders and drug-naïve patients (treatment effectiveness), and (3) non-responders and drug-naïve patients (treatment resistance). Given sites are sorted in the decreasing order of average methylation difference (delta beta (Δβ)).

DMR analysis identified 1,072 regions (mapped to 1,176 genes), characterized by at least seven significantly different probes between the targeted groups, and 96.08% (1030) of these DMRs were independently replicated through DMR analysis using DMRcate (**Supplementary Table S6**). *CTNNA2* (32 probes) and *IGF2* (53 probes) were the most represented regions, harboring three distinct DMRs each. Notably, the most significant DMR, located distally intergenic to *TBX3*, harbored 55 DMPs. The majority of identified regions (97.29%) reside within gene promoters, and 12.24% of the genes associated with these DMRs have been previously implicated in schizophrenia susceptibility.

#### 3.1.2 Identifying the markers for treatment effectiveness

To elucidate the methylomic underpinnings of antipsychotic efficacy and symptom improvement, a comparative analysis of DNA methylation profiles between drug-responsive and drug-naïve patients was performed. After covariate adjustment for family history, age, Sex, CD8T and neutrophil cell composition differential methylation was identified in 4,659 sites (FDR < 0.05) (**Figures 3A, B**, **Supplementary Table S7**). The estimated inflation factor and bias were 1.5 and −0.074, respectively (**Supplementary Figure S5**). Eleven CpG sites remained significant after the Bonferroni correction (p-value <0.1), and the top three significant sites were cg06669598 (*ECHDC1*, 3′UTR, Δβ = −0.17, Bonferroni adj. p-value = 0.02), cg17074213 (*TGFBR3*, 1stExon, Δβ = 0.07, Bonferroni adj. p-value = 0.04), and cg18415585 (intergenic, Δβ = 0.05, Bonferroni adj. p-value = 0.05) (**Figures 3C–E**). Within the singificant DMPs, 592 sites showed average DNA methylation difference exceeding the threshold (|Δβ| > 5%). These highly variable sites were mainly positioned within the open sea region with 46.11% and 92.06% of them displaying reduced DNA methylation in responders (**Figure 3B**). The hypermethylated probe cg01132696 in the gene body of *HLA-DPB1* exhibited the greatest mean methylation change (|Δβ| = 0.14), followed by cg25858983 (*UBTF*, |Δβ| = 0.14) and cg10861005 (*RGL2*, |Δβ| = 0.12) (**Supplementary Figures S6A–C**). Conversely, cg06669598 in the 3′UTR of *ECHDC1* was identified as the most varied hypomethylated site, followed by cg06579481 (intergenic, |Δβ| = 0.16) and cg08938155 (*TBCA*, |Δβ| = 0.15) (**Supplementary Figures S6D–F**).

In a comparative analysis between drug-responsive and drug-naïve individuals, 1,381 DMRs were identified, and 1,450 (95.24%) of these regions were reproduced by independent DMR analysis using DMRcate (**Supplementary Table S8**). Among the significant DMRs, the top three regions were located in the *BRD2* (62 DMPs), *KLLN* (50 DMPs), and *MRPS18B/PPP1R10* (35 DMPs) genes. Effectiveness-related regions were annotated to 1,929 genes, with 142 (7%) previously implicated as schizophrenia susceptibility loci in the GWAS catalog, suggesting a potential link between differential methylation and genetic predisposition to schizophrenia in the context of antipsychotic response.

#### 3.1.3 Identifying the markers for treatment resistance

Differential methylation analysis between non-responders and drug-naïve individuals identified 7,928 CpG sites (**Figures 4A, B**, **Supplementary Table S9**) with significant differences in DNA methylation levels (FDR < 0.05) with estimated inflation of 1.5 and bias −0.016 after controlling for the covariates sex, family history, neutrophil, CD4T, CD8T, NK, and B-cell count (**Supplementary Figure S7**). However, only 88 sites were found significant after the Bonferroni correction (p-value <0.1), and the top three sites were the following: cg22118655 (*ELFN2*, TSS200, Δβ = 0.05, Bonferroni adj. p-value = 1.57E−03), cg11415852 (*SORBS1*, 5′UTR, Δβ = −0.05, Bonferroni adj. p-value = 3.01E−03), and cg08213398 (*SWAP70*, Body, Δβ = −0.09, Bonferroni adj. p-value = 6.26E−03) (**Figures 4C–E**). A large share of the sites (n = 1,118) with average DNA methylation levels greater than 0.05 (n = 1,161) displayed decreased DNA methylation in non-responders. These resistance DMPs were dispersed across the open sea region, occupying 50.04% of the identified loci (**Figure 4B**). Moreover, these DMPs showed enrichment within the gene body (35.75%) and chromosome 1 (10.51%). *RGL2* harbored the most varied hypermethylated probe, cg10861005, with a |Δβ| of 0.12 (**Table 2**), followed by cg26220594 (intergenic, |Δβ| = 0.12) and cg12697442 (*YAP1* |Δβ| = 0.1) (**Supplementary Figures S8A–C**). Likewise, hypomethylated probes cg00863893 (*TIMP2*, |Δβ| = 0.24), cg10317314 (intergenic, |Δβ| = 0.19), and cg14687298 (intergenic, |Δβ| = 0.18) displayed the highest between-group differences in the DNA methylation level (**Supplementary Figures S8D–F**). *C13orf26* harbored the most number of hypomethylated sites within its coordinates (n = 5), followed by *SLC17A9* (n = 4), *PTPRN2* (n = 4), *ARHGEF10*, *TNXB*, *ADAM5P*, *C6orf10*, *PKP3*, and *SDK1* (n = 3 each).

Differential methylation analysis revealed 892 resistance-related regions mapped to 1,211 genes, with 97.53% (n = 870) of these regions being replicated through independent analysis using DMRcate (**Supplementary Table S10**). The top three significant regions were annotated to *FAM132A*, *BRD2*, and *HOXA4*, while the resistance DMRs containing a maximum number of CpG probes were represented by genes such as *BRD2* (n = 47), *BAT2* (n = 33), *GPR75-ASB3* (n = 30), *HLA-DPB2* (n = 30), and *LOC100302652* (n = 30). Furthermore, three differentially methylated regions were reported in the *BAT2* gene. Interestingly, only 82 of the DMR genes have been previously implicated as schizophrenia risk loci.

#### 3.1.4 Gene ontology

**Figure 5:**
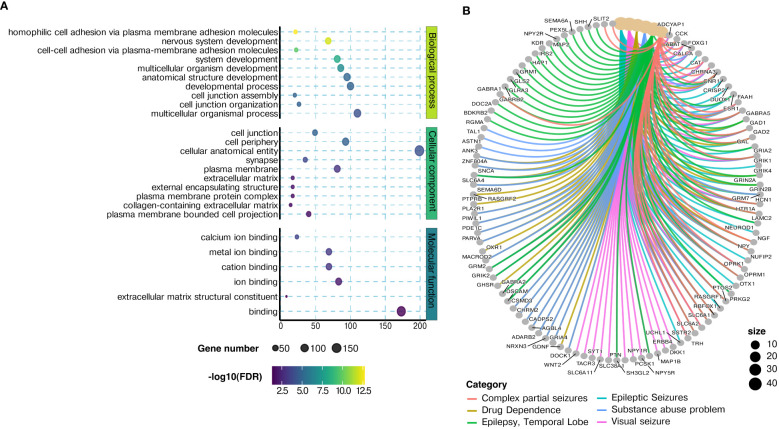
Gene ontology terms related to differentially methylated probes. **(A)** Bubble graph of the top 10 ontology terms enriched for the significant differentially methylated sites with |Δβ| ≥ 5% between the responders and non-responders. The X-axis denotes the number of differentially methylated genes associated with gene ontology (GO) terms, and the Y-axis displays the GO terms of the categories: biological process (BP), cellular component (CC), and molecular function (MF). The color of the bubble denotes the −log10 (p-value) of the significance of enrichment, and the size represents the count of differentially methylated probe (DMP) genes. **(B)** Cnet plot of ontology terms enriched with a p-value <0.05 after differentially methylated region (DMR) gene–disease association enrichment analysis. Top six ontology terms and the genes associated with each term are depicted in the circular plot, and the node size denotes the number of genes in each category.

Gene ontology analysis of DMPs for the treatment response group identified enrichment for cellular processes linked to cell adhesion, nervous system development, and cell-to-cell signaling (**Figure 5A**, **Supplementary Table S11**). In contrast, the DMR genes are enriched for processes such as neuron differentiation, pattern specification, regionalization, and specific KEGG pathways including “neuroactive ligand-receptor interaction” (FDR = 4.93E−15) and “axon guidance” (FDR = 0.02) (**Supplementary Table S12**). Additionally, an overrepresentation of disease–gene networks pertaining to drug dependence and substance-related disorders was observed for the DMR genes (**Figure 5B**, **Supplementary Table S13**).

Gene ontology analysis of DMPs for treatment effectiveness identified enrichment for terms connected to various cellular processes involved in cellular communication and its regulation (**Supplementary Table S14**), while the key ontology terms related to DMR-associated genes included RNA catabolism, ribosome subunit synthesis, arrangement, and assembly, suggesting a potential role of altered epigenetic regulation in these processes contributing to treatment effectiveness (**Supplementary Table S15**). Similarly, pathway analysis revealed a significant association between DMRs linked to effective antipsychotic response and genes involved in the ribosome pathway (FDR = 7.59E−03).

**Figure 6:**
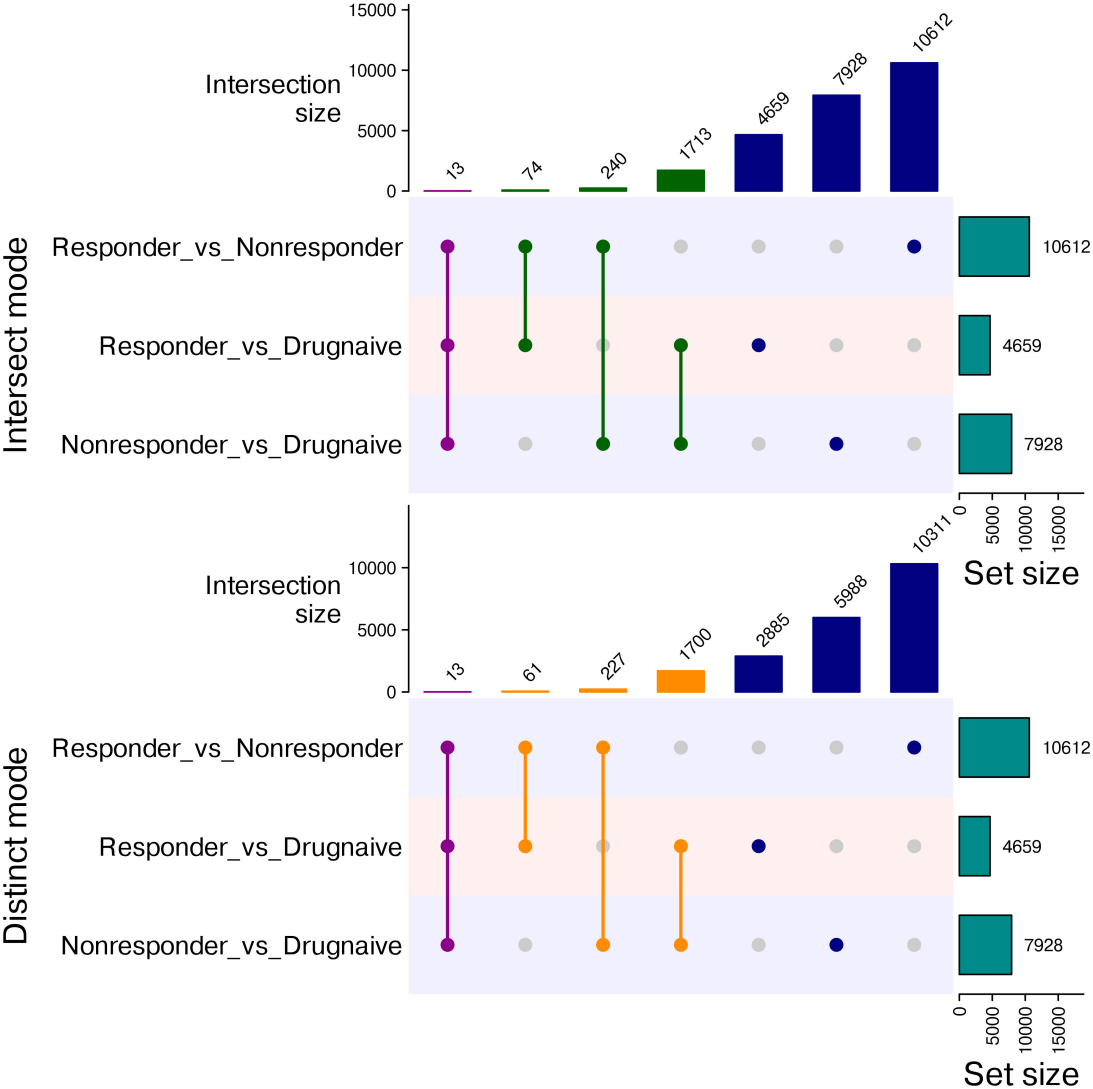
Overlapping differentially methylated sites between various comparisons. Upset plot showing the number of overlapping and distinct CpG sites in the comparisons between (1) responders and non-responders, (2) responders and drug-naïve patients, and (3) non-responders and drug-naïve patients; the bar graph on the side shows the total number of differentially methylated probes (DMPs) in each comparison.

Gene ontology analysis of DMPs for the treatment resistance group displayed enrichment for processes related to DNA methylation, miRNA metabolism, and its regulation (**Supplementary Table S16**). Enrichment of pathways such as metabolism of riboflavin (p-value = 8.95E−04) and biosynthesis of mucin-type O-glycan (p-value = 2.21E−02) was found unique to resistance DMPs, although it was not significant after FDR correction. Additionally, the resistance DMRs displayed enrichment of pathways related to spliceosomes (FDR = 5.53E−03) and GO terms like DNA synthesis, non-coding RNA processing, and metabolism of RNA, rRNA, and piRNA (**Supplementary Table S17**). Several sites were observed to overlap across the comparisons, as depicted in the Upset plot (**Figure 6**), and this was reflected in the overlap between GO terms (FDR > 0.05) between the resistance DMPs and treatment-effective DMPs (overlapping p-value = 2.7e−138, Jaccard index = 0.3). The shared list of terms includes cell adhesion, communication, and regulation.

#### 3.1.5 Protein–protein interaction network

STRING analysis of protein encoded by treatment response DMR genes revealed a high degree of connectivity; therefore, Cytoscape plugin MCODE was used to identify subnetworks with a combined score >0.6, and two distinct clusters centered around proteins associated with GABAergic signaling: GABRA4 (GABA receptor subunit) and GAD1 (glutamate decarboxylase enzyme) with node score >5 were identified.

The DMPs of treatment effectiveness formed a single network, with the ribonucleoprotein SNRPD3 acting as the central seed protein. In contrast, DMR-associated proteins formed 12 distinct modules with ribosomal protein RPS6, nucleosomal protein CENPA, and transcription coactivator protein MED22, serving as the seed for the top clusters.

**Figure 7:**
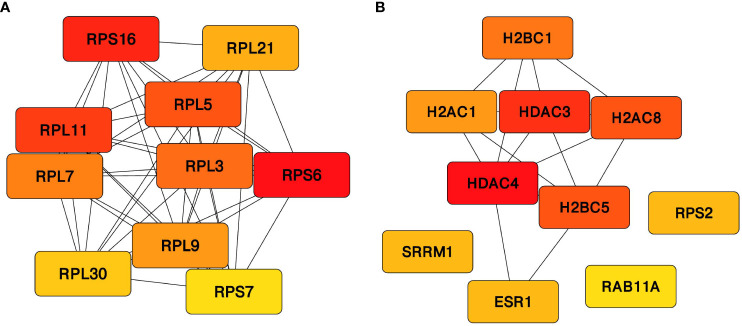
Hub proteins. Top 10 hub proteins identified from the unique genes associated with the regions differentially methylated between the **(A)** responder and drug-naïve patients and **(B)** non-responder and drug-naïve patients using Maximal Clique Centrality (MCC) algorithm. Directionality of the ranking is represented by the color gradient ranging from red (ranked first) to yellow.

Similarly, treatment resistance DMPs formed a single cluster with ribosomal protein RPL3 as seed. However, DMR proteins formed nine clusters, and the top three were centered around ribonuclease RPP38, mismatch repair protein MLH1, and ATP5PF. CytoHubba was used to identify and rank the hub proteins from proteins encoded by DMP and DMR: DMR-associated genes were unique to effectiveness and resistance (**Figures 7A**, **B**). Notably, ribosomal proteins RPS6, RIL16, and RPL11 were recognized as the hub protein among the effectiveness markers. Conversely, histone modifiers HDAC4, HDAC3, and nucleosome component H2BC5 were ranked in the top three positions connected to resistance DMR markers. The results of the protein–protein interaction (PPI) analysis of all DMP- and DMR-associated genes for all three comparisons are given in **Supplementary Table S18**.

### 3.2 Methylation quantitative trait loci associated with treatment response based on GSA

To investigate variants that influence DNA methylation signatures and modulate treatment response, we performed a meQTL analysis and identified 23 *cis* and *trans* CpG–SNP pairs (FDR ≤ 0.1) associated with the significant DMPs related to the antipsychotic treatment response. We demonstrated that based on SNPs mapped in the study, our population groups are of South Asian ancestry (**Supplementary Figure S9**). These reported *cis*-meQTLs were positioned within 50 kb of 17 significant DMPs, while the *trans*-meQTLs were linked with four CpG sites (cg07796745, cg15035716, cg03073851, and cg27380599). The two *cis*-meQTLs involved missense variants: rs36092215 (cg21934190, *GPR20*) and rs6657616 (cg08133631 and cg20415053, *CATSPER4*). Although these meQTLs were not directly associated with treatment response or schizophrenia risk, the DNA methylation level at cg22705746 (*FMOD*, TSS1500) and cg26987645 (*FMOD*, TSS200) were influenced by rs10494841, which is in LD with variants rs16851364 and rs4971252 that are reportedly associated with serum fibromodulin levels. All eQTLs related to response-related *cis*- and *trans*-DNA methylation quantitative trait loci are given in **Supplementary Tables S19**-**21**.

### 3.3 Effect of antipsychotic drugs on the mRNA level of differentially methylated genes

We investigated the expression pattern of some of the critical differentially methylated (|Δβ| ≥ 0.10) genes expressed in PBMC to study the impact of drug-induced DNA methylation. We examined their expression changes after clozapine, olanzapine, risperidone, and haloperidol treatment (**Table 3**, **Supplementary Figures S10**-**13**). We observed a varying expression pattern of the response markers *SET*, *ECHDC1*, *GET4*, *TBCA*, *ZFAND2A*, *CORO1C*, and *C7orf50* to the antipsychotic drugs. Clozapine and haloperidol treatments significantly elevated the expression of most response markers. We then checked the variance in mRNA levels of classes I histone deacetylases (*HDAC1*, *HDAC2*, and *HDAC3*), class IIa (*HDAC 9*), class IIb (*HDAC6*), and class III (*SIRT2*), histone acetylase (*KAT5*), and methyltransferases (*KMT2E*, *KDM5A*, *PRMT1*, and *PRMT5*). Olanzapine promoted concentration-dependent gene expression of all histone deacetylase genes, while risperidone normalized *HDAC* expression. Although haloperidol, clozapine, and risperidone raised *KAT5* expression, only the haloperidol treatment was significant. Similarly, haloperidol treatment introduced a significant upregulation of *KDM5A*. Clozapine, haloperidol, and olanzapine elevated the *KMT2E* transcript with increasing concentration. *PRMT1* expression increased significantly with increased concentrations of clozapine, risperidone, and olanzapine; at the same time, *PRMT5* showed a concentration-dependent increase with clozapine, olanzapine, and haloperidol treatment. We also explored the influence of antipsychotic medications on the mRNA levels of human leukocyte antigen (HLA) genes such as *HLA-A*, *HLA-B*, *HLA-C*, *HLA-E*, *HLA-DPA1*, *HLA-DPB1*, *HLA-DQA1*, *HLA-DQB1*, *HLA-DRA*, and *HLA-DMB*. The gene expression profile indicates the upregulation of class I HLA and the downregulation of class II HLA, with clozapine having the maximum impact, followed by haloperidol and risperidone.

**Table 3:** Effect of antipsychotic drugs on the mRNA level of treatment response, histone modifiers, and human leukocyte antigen genes in PBMC.

Gene name	Clozapine		Olanzapine		Risperidone		Haloperidol	
	DMSO—8 μM	2–8 μM	DMSO—3.2 μM	0.8–3.2 μM	DMSO—4 μM	1–4 μM	DMSO—12 μM	3–12 μM
*SET*	**0.0061⇑**	0.5621	**0.041⇓**	**0.0438⇓**	0.3765	0.0716	**0.0002⇑**	**0.0003⇑**
*ECHDC1*	**0.0132⇑**	**0.0252⇑**	0.2074	0.1013	0.585	**0.0027⇓**	0.1737	**0.0309⇓**
*GET4*	**0.0451⇑**	0.1911	0.1689	0.0914	0.2891	0.115	**0.0059⇑**	**0.0152⇑**
*TBCA*	**0.0005⇑**	0.1404	0.1263	**0.0421⇓**	**0.0354⇑**	0.5154	**0.0422⇑**	0.0948
*ZFAND2A*	0.8231	**0.0495⇑**	**0.0001⇑**	**0.0016⇑**	0.5993	0.0599	**0.0205⇑**	**0.0122⇑**
*CORO1C*	**0.0381⇓**	0.7834	**0.0019⇑**	**0.0005⇑**	0.5933	0.4299	**0.0013⇑**	**0.0046⇑**
*C7ORF50*	0.4129	0.4468	0.9994	0.7849	**0.0017⇑**	**0.0012⇓**	0.3913	**0.0399⇓**
*HDAC1*	**0.0154⇑**	**0.0015⇑**	**0.0034⇑**	**0.0007⇑**	0.094	**0.0096⇓**	**0.0046⇑**	0.3851
*HDAC2*	**0.0102⇑**	0.4835	0.0845	**0.0063⇑**	0.8874	**0.0143⇓**	0.9912	**0.0086⇓**
*HDAC3*	**0.0045⇑**	**0.0007⇑**	**0.0145⇑**	**0.0086⇑**	0.9996	0.1021	0.0938	0.1933
*HDAC6*	0.1745	0.**0422⇑**	0.2333	**0.0352⇑**	0.1937	0.0945	**0.0115⇑**	0.0969
*HDAC9*	0.279	**0.0057⇓**	**0.0002⇑**	**<0.0001⇑**	0.8172	0.4035	**0.047⇓**	**0.0015⇓**
*SIRT2*	0.5553	0.5798	**0.0177⇑**	**0.0169⇑**	0.9999	0.5929	0.1556	0.1417
*KAT5*	0.535	0.1048	0.6002	>0.9999	0.846	0.7654	**0.031⇑**	0.1775
*KMT2E*	**0.0128⇑**	**0.0286⇑**	0.0823	**0.0403⇑**	0.1479	0.1702	**0.0059⇑**	**0.009⇑**
*KDM5A*	0.547	0.0876	0.0732	0.2065	0.2307	0.2372	**0.0017⇑**	**0.0099⇑**
*PRMT1*	**0.0496⇑**	**0.0248⇑**	**0.0055⇑**	**0.0006⇑**	0.1913	**0.0418⇑**	0.2472	0.6262
*PRMT5*	0.4214	**0.0345⇑**	**0.0067⇑**	**0.001⇑**	0.5992	**0.0315⇑**	**0.0026⇑**	**0.0154⇑**
*HLA-A*	**0.0069⇑**	**0.0008⇑**	0.0697	**0.0356⇑**	0.3602	0.4206	**0.0002⇑**	**0.0004⇑**
*HLA-B*	**0.0013⇑**	**0.0116⇑**	0.2867	**0.0186⇑**	0.996	>0.9999	**0.0482⇑**	**0.0179⇓**
*HLA-C*	0.3264	**0.0004⇑**	0.9366	0.2971	0.9992	0.5644	**0.0058⇑**	**0.0087⇑**
*HLA-E*	0.1104	**0.038⇑**	0.3177	0.2612	0.9843	0.5392	0.7506	0.5016
*HLA-DPA1*	0.7879	**0.0146⇓**	0.4864	0.2995	0.2107	0.0704	**0.0487⇓**	0.7507
*HLA-DPB1*	**0.0382⇓**	0.7013	0.4493	0.9769	0.1465	**0.0498⇓**	**0.0214⇓**	**0.0243⇓**
*HLA-DQA1*	**0.025⇓**	0.1052	0.8558	**0.0037⇓**	0.4541	0.948	**0.0133⇓**	0.0522
*HLA-DQB1*	**0.0076⇓**	**0.0229⇓**	0.887	0.1399	**0.0056⇓**	0.9696	**0.0435⇓**	0.593
*HLA-DRA*	**0.0009⇓**	**0.0044⇓**	0.9801	0.097	0.076	0.3553	**0.0222⇓**	0.087
*HLA-DMB*	**0.0082⇑**	0.8509	0.9793	**0.0132⇓**	0.0844	0.1065	**0.0217⇓**	**0.0057⇓**

Each cell represents p-value of the one-way ANOVA with Sidak’s multiple-comparison test of the corresponding comparison; significant p-values are displayed in bold character, up arrow and down arrow indicate gene upregulation and downregulation, respectively.

PBMC, peripheral blood mononuclear cell.

## 4 Discussion

The current study represents the first genome-wide DNA methylation study ever conducted in an Indian population aimed at understanding the epigenetic markers of schizophrenia pathology and distinguishing them from markers of therapeutic response and resistance. Antipsychotic drugs target neurotransmitter pathways and mediate therapeutic responses. However, the lack of consistent observations and failure to relate response to genetic variants in these neurotransmitter pathways (57) strongly indicate alternate mechanisms, like epigenetics, for therapeutic response. The previous epigenetic studies employed peripheral blood or post-mortem samples from patients with conventional treatment backgrounds, potentially influencing the identification of pathogenesis-related epigenetic markers due to the modulatory effect of antipsychotics on DNA methylation (58). In a clinical scenario, it is difficult to control the medication and test for its impact on the host epigenome; therefore, to overcome this challenge, we conducted methylome-wide studies on schizophrenia patients by stratifying them into responders, non-responders, and drug-naïve patients. The study identified several unique DMPs and DMRs as methylomic markers for drug response, effectiveness, and refractoriness.

We investigated the impact of conventional treatment protocol on the methylome and found that the state of hypermethylation is largely implicated in drug response and hypomethylation with drug resistance or non-responsiveness. This was also evident in an earlier observation based on the overexpression of DNMT genes and its resultant global hypermethylation upon antipsychotic treatment (24). The methylome-wide observations on treatment responder and non-responder groups when compared with previous studies on case–control DNA methylation variations (18, 20, 22, 59–61) had contrasting and conflicting DNA methylation patterns (hyper and hypo) and the direction of DNA methylation difference (hypo/hyper or hyper/hypo) (**Supplementary Table S22**). Therefore, it is important to carefully assess the medication-induced methylomic alterations for their favorable effect on etiology, course of illness development, intensity of symptoms, adverse reactions, and refractoriness.

A comprehensive understanding of the extent of differences between responder and non-responder DNA methylomes is necessary to depict treatment response heterogeneity accurately. The most significant probe identified in the study was localized to *LBX1*, a transcription factor encoding gene that is essential for GABAergic differentiation in the dorsal spinal cord (62) and midbrain dopaminergic neuron specification, potentially through the regulation of cholesterol biosynthesis (63). Another significant probe was localized to *A2BP1*, which is a schizophrenia risk gene and reported to have a reduced expression in the cortical regions of schizophrenia patients (64) and genetic association with olanzapine-induced weight gain in the Chinese Han population (65), suggesting its involvement in both pathogenesis and treatment response. The most significant hypermethylated response-linked DMP was found within the *MIER2* gene, encoding a nuclear protein that recruits HDACs (66). Interestingly, *MIER2* hypomethylation has been reported in relation to fetal membrane infection (67). In addition, traits associated with hypermethylated response DMPs hint at the significance of early environmental insults occurring at the gestational stages or during childhood in shaping DNA methylation patterns and heightening the susceptibility to schizophrenia. We observed multiple response-related probes aligning with genes involved in neuronal development and function, including *FILIP1*, *PM20D1*, and *GALNT9*. Hypermethylation of FILIP1, a filamin A-binding protein regulating cortical neuron migration and dendritic spine morphology, is significant, as it could impair these major processes (68, 69). Similarly, *PM20D1*, involved in the amide biosynthesis and regulation of neuron death, has been related to enhanced glucose homeostasis in mice (70) and also DNA methylation changes in *PM20D1* linked to PTSD and Alzheimer’s disease (71, 72). Another top-hit hypermethylated gene, *GALNT9*, has been implicated in schizophrenia through glycosylation dysregulation (73). Interestingly, first-episode schizophrenia patients undergoing risperidone treatment reported differential methylation in the gene region involved with O-linked glycosylation; also, first-episode drug-naïve schizophrenia-associated differential methylation marker cg15150970 was replicated in our study between responders and non-responders (74). Several of the peripheral tissue-based DNA methylation patterns associated with treatment response were found to parallel the brain pattern (**Supplementary Table S23**).

Several cell adhesion molecules belonging to the protocadherin cluster were found to be differentially methylated between responsive and non-responsive subjects. Cell adhesion molecules coordinate the trafficking of immune components across the blood–brain barrier, which is deregulated in psychosis conditions (75, 76). We have reported the involvement of proinflammatory factors in schizophrenia (77), and interestingly, antipsychotics are known to influence the immune response (78). The association of DNA methylation differences around the cell adhesion molecule cadherin with treatment response stresses the role of antipsychotic-mediated regulation of peripheral inflammation and its long-range effect on the neuronal tissues. It has been reported that cadherin expression can be regulated by the hsa-miR-29b-3p/DNMT3b/MMP-9 pathway in human brain microvascular endothelial cells (79), and interestingly, miR-29b and DNMT expression have been reported to be influenced by antipsychotic drugs (24).

Our analysis identified multiple DMRs mapping to *IGF2* and *CTNNA2. CTNNA2*, encoding an actin regulator, α-catenin, is important for neuronal migration and neurite length (80), and an intronic variant within this gene serves as a biomarker for lurasidone response in the European population (81). Moreover, DNA methylation difference in the *CTNNA2* probe has also been reported in monozygotic twins discordant for psychosis (82). These findings suggest that while certain genetic markers are significant, the same gene might still be affected by an epigenetic trade-off, functioning as a substitute even in the absence of such markers. Association between *IGF2* enhancer hypomethylation in prefrontal cortex neurons and increased tyrosine hydroxylase protein has been established in schizophrenia and bipolar patients, indicating epigenetic regulation of dopamine synthesis through *IGF2* methylation (83). This aligns with the reports of IGF2 downregulation in the dorsolateral prefrontal cortex (84) and lower IGF2 serum levels in schizophrenia patients (85). In addition, the serum level of IGF2 is negatively correlated to the negative symptoms in patients while being positively associated with working memory and executive function, highlighting the importance of IGF2 in memory dysfunction (85).

The top hits on the GO terms for response indicated the role of synaptic interaction, glycosylation, extracellular matrix components, histone modifications, and cell-to-cell adhesion; interestingly, these cellular components were also reported to be implicated in schizophrenia (12, 86). Our findings revealed a directional overlap between pathogenesis and therapeutic response when they were compared over previously reported pathogenesis markers, indicating the necessity for a rigorous assessment of the DNA methylation markers of pathogenesis for the epigenetic indicators of treatment response. Understanding the trade-offs between genetic and epigenetic findings is also necessary.

We observed increased DNA methylation in drug-naïve subjects, while individuals under medications displayed reduced DNA methylation, aligning with previous studies (23). Drug-mediated changes in the methylome can have diverse on- or off-target effects, influencing the treatment efficacy by modulating the non-target gene. Comparison between responders and drug-naïve patients revealed several off-target effects of antipsychotics with hypermethylation at sites within *HLA-DPB1*, *UBTF*, and *RGL2* in responders. The *HLA-DPB1* has been linked to clozapine-induced agranulocytosis (87, 88). The most significant hypomethylated sites were observed in metabolic repair enzyme, *ECHDC1*, and a transmembrane proteoglycan encoding gene *TGFBR3*, which is known to take part in neuronal differentiation (89). Earlier studies in the blood of schizophrenia patients have identified DMPs within *TGFBR3* (90) and reported its downregulation (91). The hypermethylation status of *HLA-DPB1* and *TGFBR3* may indicate a positive response, while *ECHDC1* hypomethylation specifies metabolic stress upon antipsychotic use. Therefore, careful monitoring of antipsychotics can help differentiate the positive and negative effects of immunomodulatory and metabolic events.

While assessing the differential DNA methylation markers between the drug-naïve and non-responders, we observed hypomethylation as a dominant signature associated with treatment resistance, with genes like *ELFN2*, *SORBS1*, *SWAP70*, *PTPRN2*, and *TIMP2* being the top hits. Interestingly, *ELFN2*, a postsynaptic cell adhesion molecule, hypermethylated in non-responders has been previously linked to neuropsychiatric behavior (92). *SORBS1*, a major player in the insulin signaling pathway, displayed increased expression in schizophrenia patients with high inflammation (93, 94). Resistance-related hypomethylation in our study corroborates the damaging effects of schizophrenia risk genes *TIMP2* (95) and *SORBS1* on aggravating pathology. In contrast, SWAP70 involved in the cell adhesion suppressed inflammatory responses (96). *PTPRN2*, an inflammation marker, was reportedly found to be differentially methylated in response to risperidone treatment (74, 97). Similarly, the resistance marker cg17221813, located in the gene body of the transport protein SLC17A9, has been associated with treatment-resistant schizophrenia (98). Collectively, this study highlights the significance of differential DNA methylation in metabolic and inflammatory pathways in driving non-response and possibly drug-induced side effects.

GO keywords related to cellular communication and regulation were repeated in treatment effectiveness and resistance comparisons, reflecting the major mechanism of drug action (99). Notably, pathway and network analyses revealed a strong correlation between DMRs associated with an effective antipsychotic response and genes implicated in the ribosome pathway, providing more evidence to support the hypothesis that treatment efficacy may be influenced by epigenetic modulation of ribosome-related genes (100). Interestingly, HDAC proteins were ranked top among resistance DMR proteins, suggesting their potential role in treatment resistance.

A few top-hit DMPs were further assessed for their impact on gene expression by grouping them into response markers, histone modifiers, and HLA markers. Interestingly, response markers and histone modifiers show increased expression in most drug treatments, while in HLA, the class I HLA markers show increased expression in sharp contrast to the decreased expression of HLA class II gene expression. Response markers like *SET* (101), *ECHDC1* (102), *GET4* (103), *TBCA* (104, 105), *ZFAND2A* (18, 106), *CORO1C* (107), and *C7orf50* (108) are known to impact schizophrenia, neuronal function, cytoskeleton dynamicity, or brain metabolism. Therefore, differential expression upon antipsychotic treatment provides new insight into the downstream effect of drug-mediated methylomic changes, and it indicates that antipsychotic drugs can reverse the pathology by influencing gene expression or impacting DNA methylation patterns. HDACs have a regulatory role in synaptic plasticity, neurodegeneration, and cognitive function (109), and decreased expression of class I HDAC has been reported in schizophrenia (110, 111). Most studies on HDAC dysregulation in the brain come from postmortem brains, unsure of the responder or non-responder category. Such observations need to be relooked, as we observed hypomethylation of *HDAC9* among the resistance markers.

In recent times, the HLA region has gained significance because of its role in schizophrenia, its comorbid conditions, and the adverse effects of antipsychotic drugs. DNA methylation analysis indicated that *HLA-DPB1* hypermethylation and HLA-E hypomethylation were largely associated with a good therapeutic response, while non-responders displayed hypomethylation in *HLA-DPB2*, *HLA-DMA*, and *HLA-DMB*. Furthermore, the gene expression profiles of HLA genes post-antipsychotic treatment indicate that class I genes are often upregulated, while class II genes are often downregulated, with clozapine having the maximum impact, followed by haloperidol and risperidone. Studies have indicated that HLA genes are also associated with schizophrenia (112–115). Certain HLA alleles, such as HLA-DRB1*04:02, HLA-DPB1*05:02, *HLA-DQB1 (126Q)*, *HLA-B (158T)*, and HLA-B*59:01, have been reported to be associated with clozapine-induced agranulocytosis (116–118). The majority of these studies were restricted to identifying genetic markers of schizophrenia or clozapine response, but possibly the DNA methylation and gene expression patterns of HLA genes may help in understanding the precise role of antipsychotics in mediating therapeutic response and distinguishing them from drug-induced side effects.

The current study is possibly the first comprehensive study on the DNA methylation signature of therapeutic response in schizophrenia, backed by genomic and gene expression profiles of some of the most critically methylated genes. Some key processes that could help us distinguish the response from non-response include alterations in neurodevelopment, immune system, ribosome biogenesis, cell-to-cell adhesion and signaling, and metabolic changes. The meQTL analysis underlines the gene–environment interplay in complex disorders to create heterogeneous phenotypes; therefore, we performed the study on a genetically and epigenetically homogenous Malayalam-speaking population to minimize the variables in a complex condition like schizophrenia. Many of the signatures listed in our study are schizophrenia risk loci, indicating that specific genetic hallmarks are essential. However, even without genetic markers, epigenetic trade-offs can influence the same gene and act as a surrogate. The study needs replication with a larger sample size and cross-validation with other ancestral and epigenetic backgrounds, environmental variables, antipsychotic use, pathology, and comorbid conditions. A precise drug-specific DNA methylation signature may further help in differentiating the individual effects of each drug on multidrug therapy that drive patients into treatment response, treatment effectiveness, and treatment resistance.

## Data availability statement

All data pertaining to the manuscript is attached in the supplementary files and additional files are also available through ZENODO repository 10.5281/zenodo.10719137.

## Ethics statement

Institutional Ethics Committee of RGCB approved the study for biomedical subjects as per the ICMR and Helsinki protocol. The studies were conducted in accordance with the local legislation and institutional requirements. The participants provided their written informed consent to participate in this study.

## Author contributions

BP: Data curation, Formal analysis, Investigation, Methodology, Validation, Visualization, Writing – original draft, Resources, Software, Writing – review & editing. NV: Data curation, Investigation, Methodology, Writing – original draft. IN: Data curation, Formal analysis, Investigation, Resources, Writing – original draft. CN: Conceptualization, Data curation, Formal analysis, Investigation, Resources, Supervision, Writing – review & editing. MB: Conceptualization, Formal analysis, Funding acquisition, Investigation, Methodology, Project administration, Resources, Supervision, Validation, Visualization, Writing – review & editing, Writing – original draft.

## Supplementary material

Supplementary Figure S1 Blood cell type comparison in each comparison Plot showing the difference in the proportion of blood cell types between various groups in each comparison, **(A)** responders and nonresponders, **(B)** responders and drug-naïve **(C)** nonresponders and drug-naïve; violin plot shows the distribution of value; and boxplot produces the summary statistics. X axis represents the type of cell population and the Y axis denotes the proportion of each cell type.

Supplementary Figure S2 QQ plot of the treatment response-related differentially methylated CpG sites before and after adjusting for inflation and bias.

Supplementary Figure S3 Boxplot of top three most significant hypermethylated **(A)** cg08617160 **(B)** cg00630212 **(C)** cg21184711 and hypomethylated **(D)** cg11309454 **(E)** cg07679219 sites between responders and nonresponders with |Δβ|≥ 5%.

Supplementary Figure S4 Boxplot of the top three most differed hypermethylated **(A)** cg20415053 **(B)** cg19637330 **(C)** cg09044981 sites between responders and nonresponders with |Δβ|≥ 5%.

Supplementary Figure S5 QQ plot of the treatment effectiveness-linked differentially methylated CpG sites before and after adjusting for inflation and bias.

Supplementary Figure S6 Boxplot of the top three most differed hypermethylated **(A)** cg01132696 **(B)** cg25858983 **(C)** cg10861005 and hypomethylated **(D)** cg06669598 **(E)** cg06579481 **(F)** cg08938155 sites between responders and drug-naïve with |Δβ|≥ 5%.

Supplementary Figure S7 QQ plot of the treatment resistance-associated differentially methylated CpG sites before and after adjusting for inflation and bias.

Supplementary Figure S8 Boxplot showing the top three most differed hypermethylated **(A)** cg10861005 **(B)** cg26220594 **(C)** cg12697442 and hypomethylated **(D)** cg00863893 **(E)** cg10317314 **(F)** cg14687298 sites between nonresponders and drug-naïve with |Δβ|≥ 5%.

Supplementary Figure S9 PCA plot of population grouping based on SNPs reflect south Asian ancestry: PCA-bi plot **(A)** principal component 1 and 2 **(B)** principal component 1 and 3.

Supplementary Figure S10
*In vitro* assessment of antipsychotic drug treatment on treatment response markers. Expression level of treatment response markers in peripheral blood mononuclear cells following the treatment of **(A)** clozapine, **(B)** olanzapine, **(C)** risperidone, and **(D)** haloperidol. Data presented as the mean ± SEM between two independent experiments (*p ≤0.05, **p ≤0.01, ***p ≤ 0.001 ****p ≤ 0.0001).

Supplementary Figure S11
*In vitro* assessment of antipsychotic drug treatment on histone acetylation and deacetylation genes. Expression level of histone acetylation and deacetylation genes in peripheral blood mononuclear cells following the treatment of **(A)** clozapine, **(B)** olanzapine, **(C)** risperidone, and **(D)** haloperidol. Data presented as the mean ± SEM between two independent experiments (*p ≤0.05, **p ≤0.01, ***p ≤ 0.001 ****p ≤ 0.0001).

Supplementary Figure S12
*In vitro* assessment of antipsychotic drug treatment on histone methylase and demethylase genes. Expression level of histone methylase and demethylase genes in peripheral blood mononuclear cells following the treatment of **(A)** clozapine, **(B)** olanzapine, **(C)** risperidone, and **(D)** haloperidol. Data presented as the mean ± SEM between two independent experiments (*p ≤0.05, **p ≤0.01, ***p ≤ 0.001 ****p ≤ 0.0001).

Supplementary Figure S13
*In vitro* assessment of antipsychotic drug treatment on Human Leukocyte Antigen genes. Expression level of Human Leukocyte Antigen genes in peripheral blood mononuclear cells following the treatment of **(A)** clozapine, **(B)** olanzapine, **(C)** risperidone, and **(D)** haloperidol. Data presented as the mean ± SEM between two independent experiments (*p ≤0.05, **p ≤0.01, ***p ≤ 0.001 ****p ≤ 0.0001).
