# Supplementary figures and images for: Methylome-wide and meQTL analysis helps to distinguish treatment response from non-response and pathogenesis markers in schizophrenia
Source: Front Psychiatry. 2024 Mar 7;15:1297760. doi: 10.3389/fpsyt.2024.1297760 (PMC10954811; doi:10.3389/fpsyt.2024.1297760)

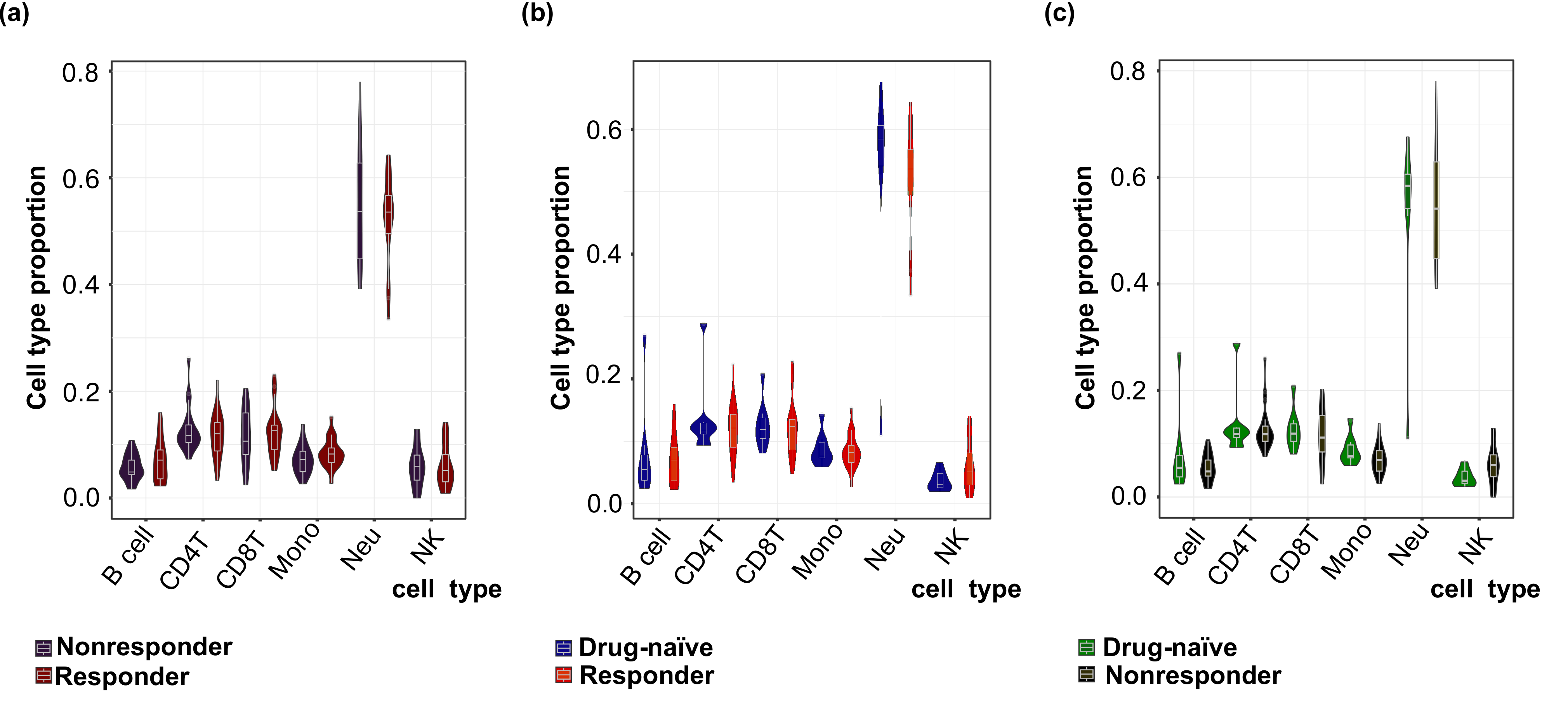

Supplement: Supplementary Figure S1 — Blood cell type comparison in each comparison Plot showing the difference in the proportion of blood cell types between various groups in each comparison, (A) responders and nonresponders, (B) responders and drug-naïve (C) nonresponders and drug-naïve; violin plot shows the distribution of value; and boxplot produces the summary statistics. X axis represents the type of cell population and the Y axis denotes the proportion of each cell type. [file Image_1.tif]

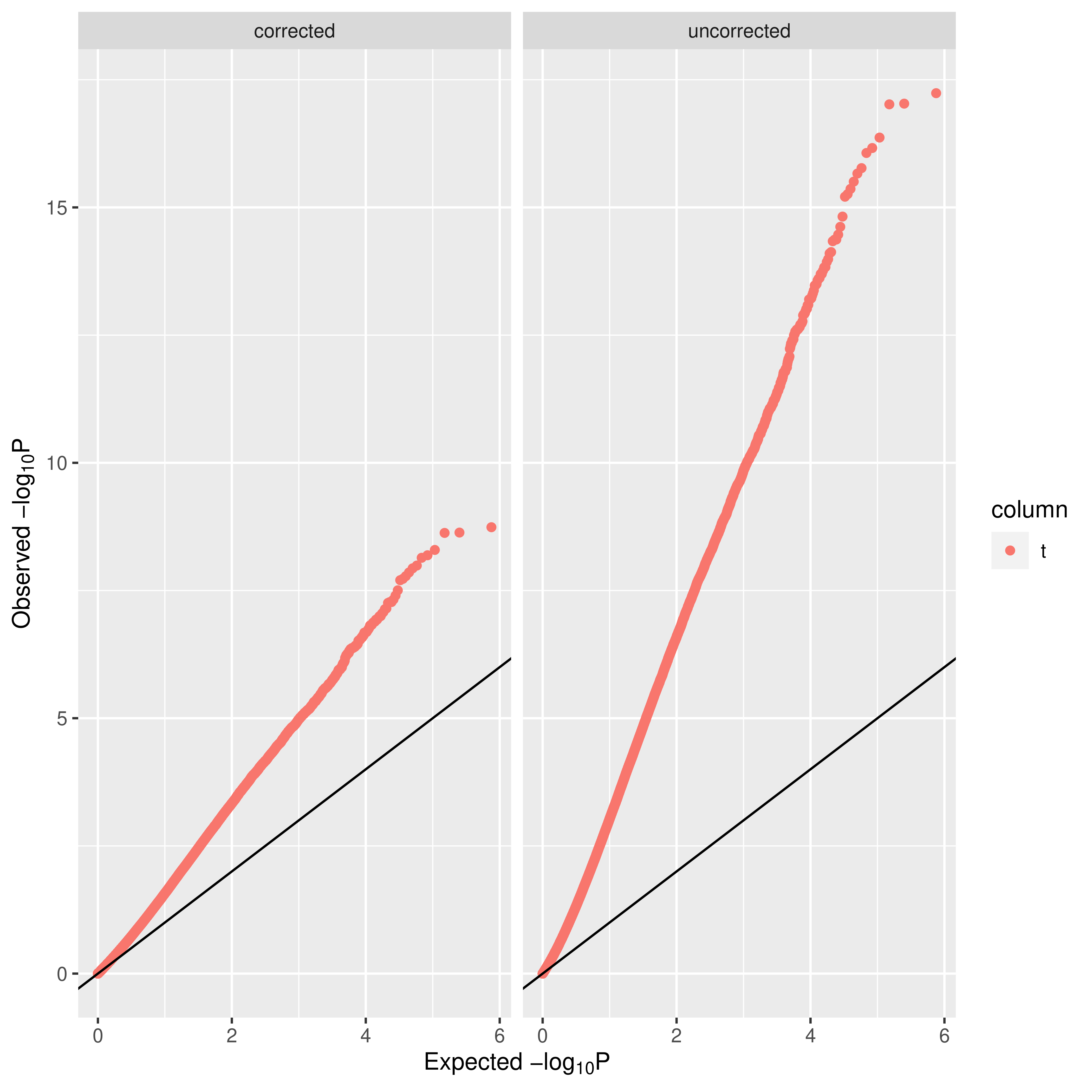

Supplement: Supplementary Figure S2 — QQ plot of the treatment response-related differentially methylated CpG sites before and after adjusting for inflation and bias. [file Image_2.tif]

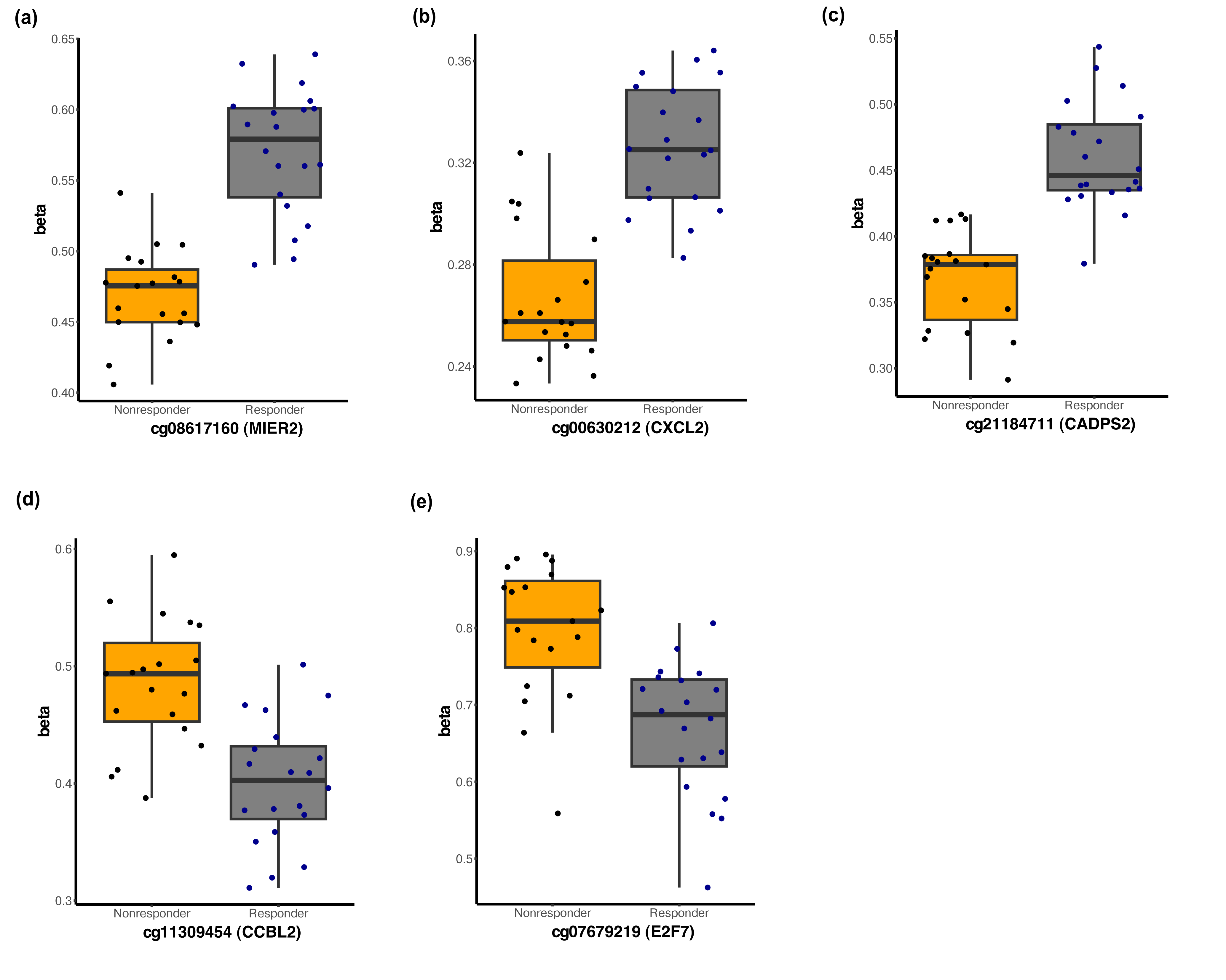

Supplement: Supplementary Figure S3 — Boxplot of top three most significant hypermethylated (A) cg08617160 (B) cg00630212 (C) cg21184711 and hypomethylated (D) cg11309454 (E) cg07679219 sites between responders and nonresponders with |Δβ|≥ 5%. [file Image_3.tif]

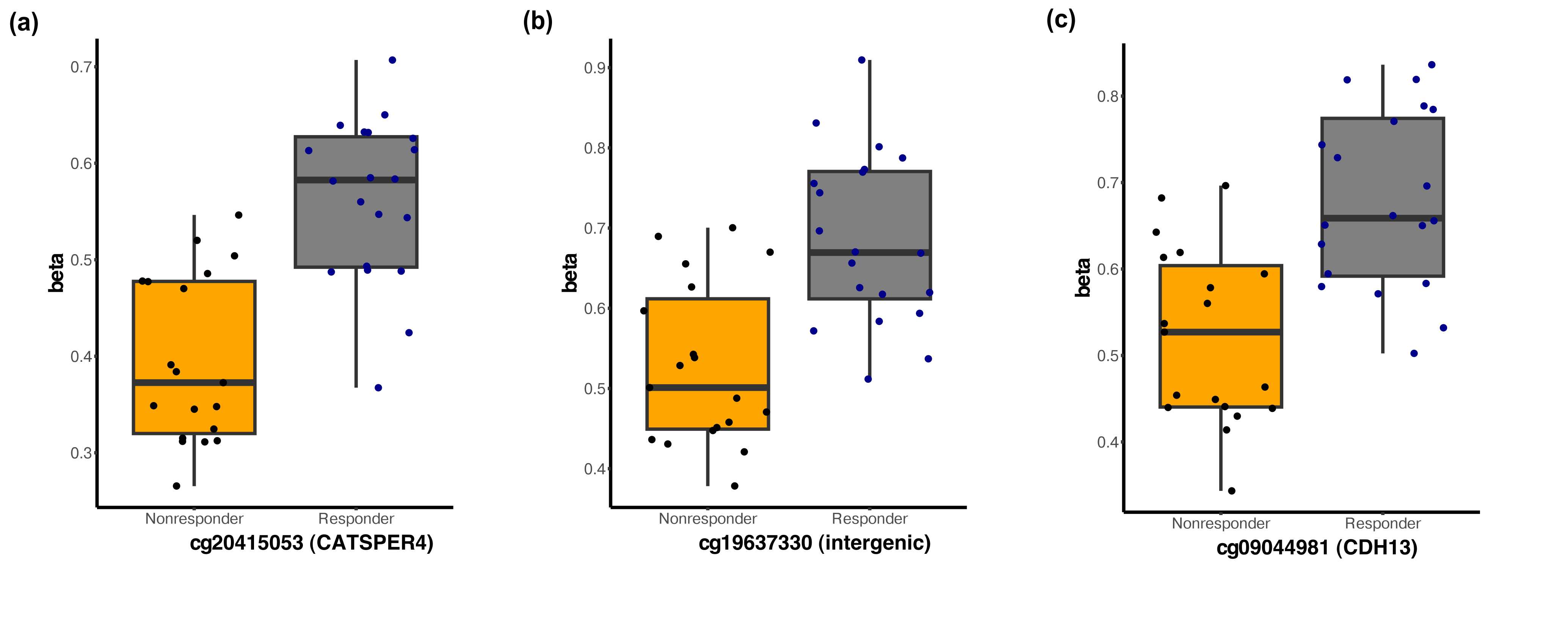

Supplement: Supplementary Figure S4 — Boxplot of the top three most differed hypermethylated (A) cg20415053 (B) cg19637330 (C) cg09044981 sites between responders and nonresponders with |Δβ|≥ 5%. [file Image_4.tif]

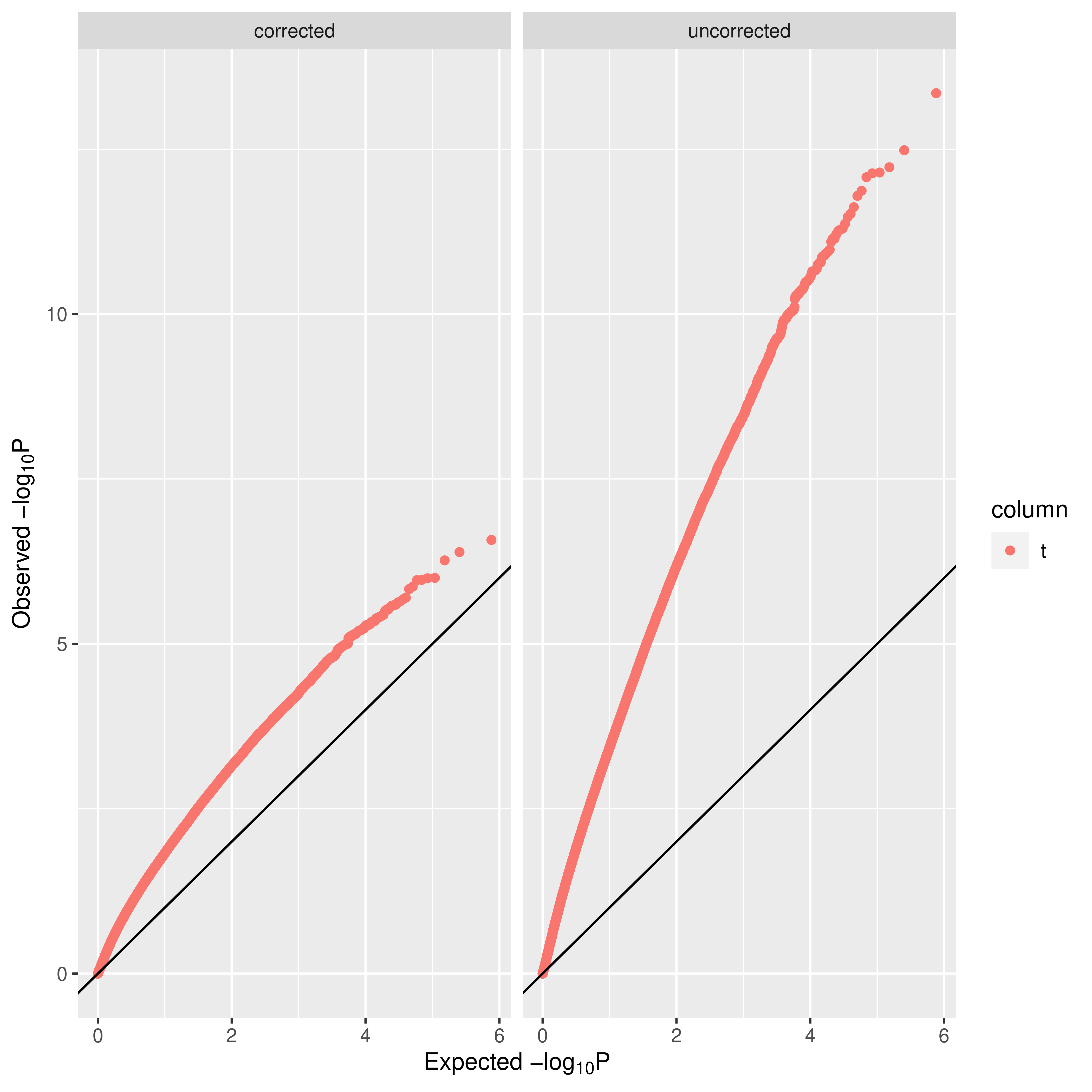

Supplement: Supplementary Figure S5 — QQ plot of the treatment effectiveness-linked differentially methylated CpG sites before and after adjusting for inflation and bias. [file Image_5.tif]

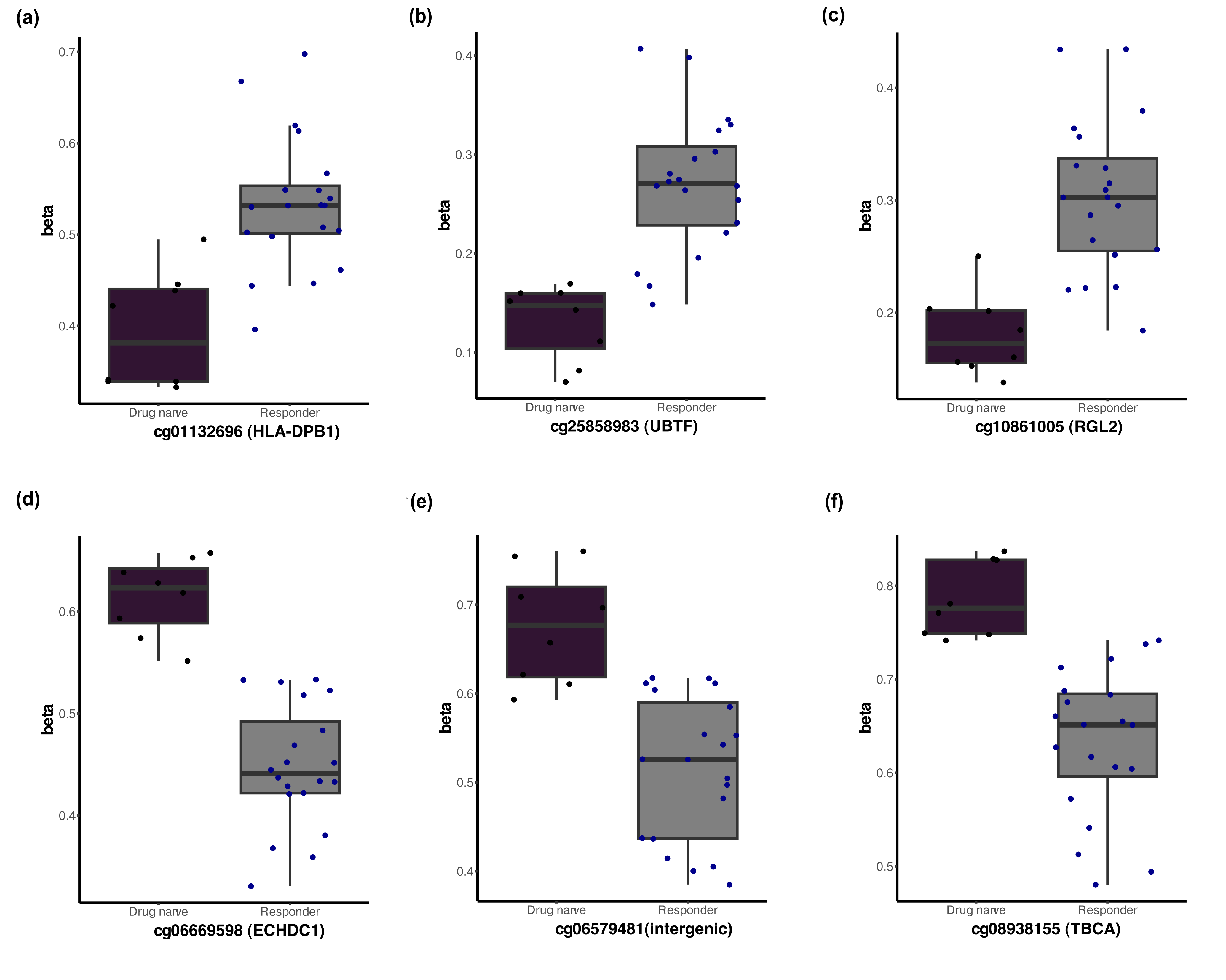

Supplement: Supplementary Figure S6 — Boxplot of the top three most differed hypermethylated (A) cg01132696 (B) cg25858983 (C) cg10861005 and hypomethylated (D) cg06669598 (E) cg06579481 (F) cg08938155 sites between responders and drug-naïve with |Δβ|≥ 5%. [file Image_6.tif]

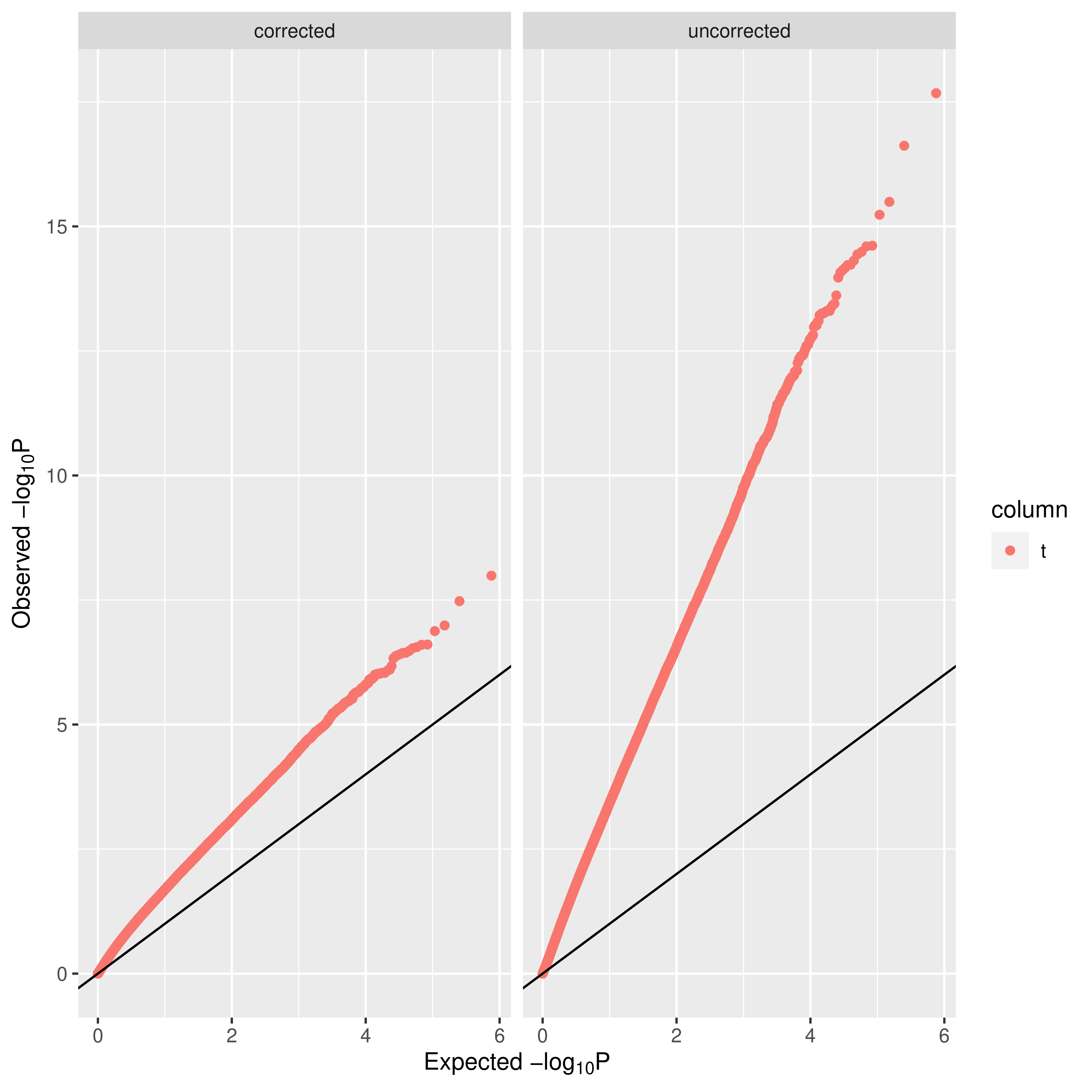

Supplement: Supplementary Figure S7 — QQ plot of the treatment resistance-associated differentially methylated CpG sites before and after adjusting for inflation and bias. [file Image_7.tif]

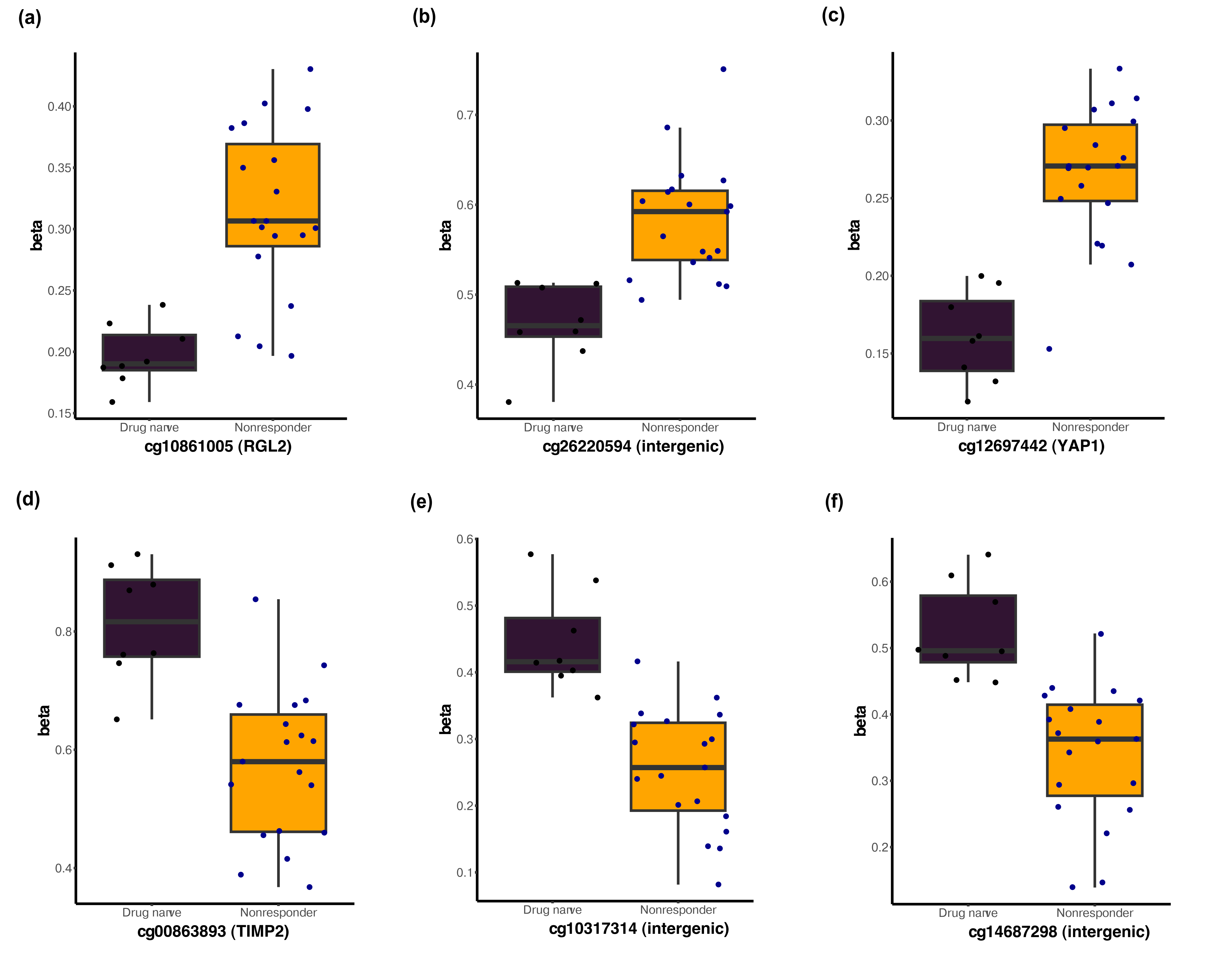

Supplement: Supplementary Figure S8 — Boxplot showing the top three most differed hypermethylated (A) cg10861005 (B) cg26220594 (C) cg12697442 and hypomethylated (D) cg00863893 (E) cg10317314 (F) cg14687298 sites between nonresponders and drug-naïve with |Δβ|≥ 5%. [file Image_8.tif]

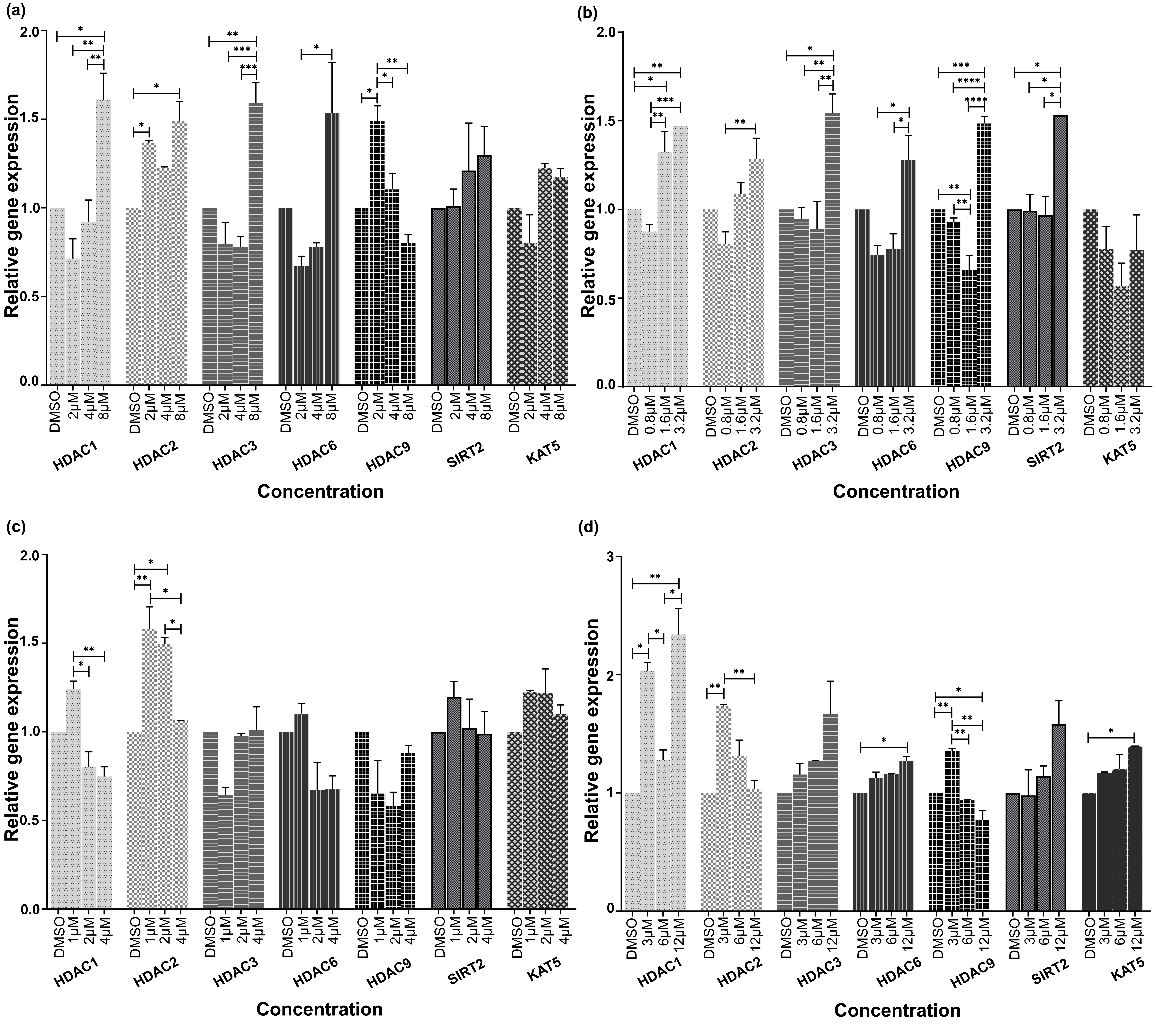

Supplement: Supplementary Figure S11 — In vitro assessment of antipsychotic drug treatment on histone acetylation and deacetylation genes. Expression level of histone acetylation and deacetylation genes in peripheral blood mononuclear cells following the treatment of (A) clozapine, (B) olanzapine, (C) risperidone, and (D) haloperidol. Data presented as the mean ± SEM between two independent experiments (*p ≤0.05, **p ≤0.01, ***p ≤ 0.001 ****p ≤ 0.0001). [file Image_11.tif]

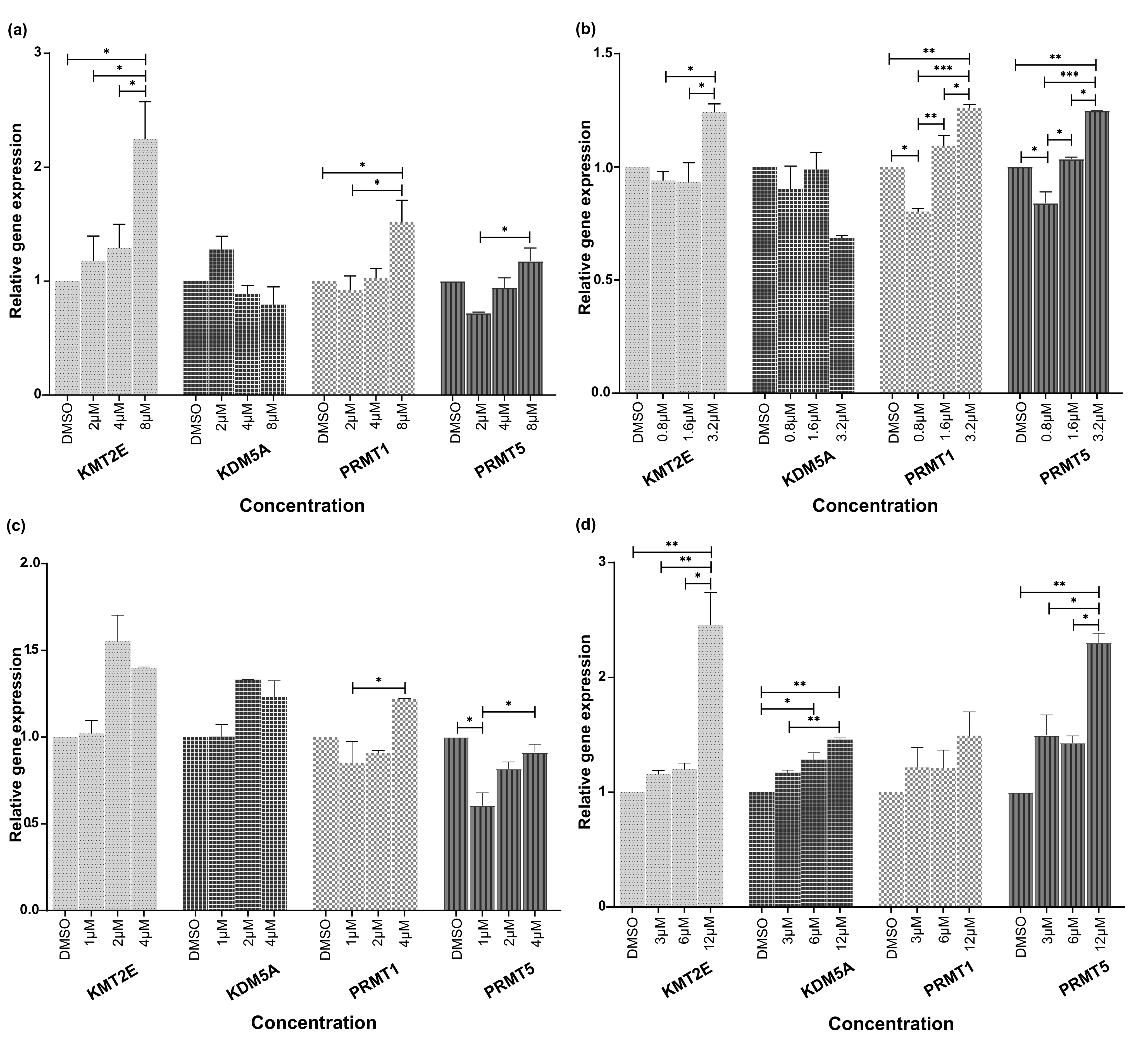

Supplement: Supplementary Figure S12 — In vitro assessment of antipsychotic drug treatment on histone methylase and demethylase genes. Expression level of histone methylase and demethylase genes in peripheral blood mononuclear cells following the treatment of (A) clozapine, (B) olanzapine, (C) risperidone, and (D) haloperidol. Data presented as the mean ± SEM between two independent experiments (*p ≤0.05, **p ≤0.01, ***p ≤ 0.001 ****p ≤ 0.0001). [file Image_12.tif]

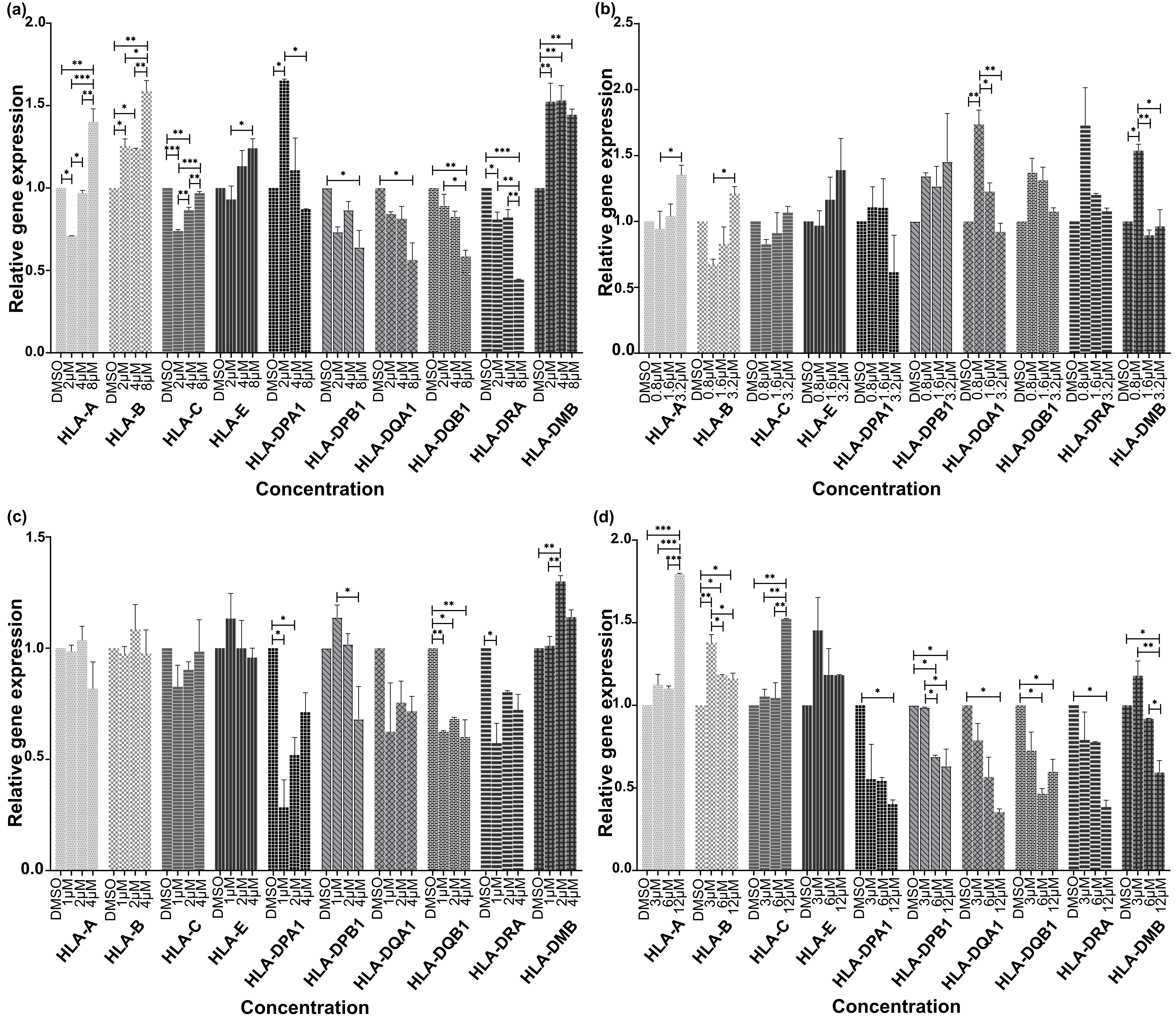

Supplement: Supplementary Figure S13 — In vitro assessment of antipsychotic drug treatment on Human Leukocyte Antigen genes. Expression level of Human Leukocyte Antigen genes in peripheral blood mononuclear cells following the treatment of (A) clozapine, (B) olanzapine, (C) risperidone, and (D) haloperidol. Data presented as the mean ± SEM between two independent experiments (*p ≤0.05, **p ≤0.01, ***p ≤ 0.001 ****p ≤ 0.0001). [file Image_13.tif]
